# Genetic Diagnosis of Charcot-Marie-Tooth Disease in a Population by Next-Generation Sequencing

**DOI:** 10.1155/2014/210401

**Published:** 2014-06-16

**Authors:** Helle Høyer, Geir J. Braathen, Øyvind L. Busk, Øystein L. Holla, Marit Svendsen, Hilde T. Hilmarsen, Linda Strand, Camilla F. Skjelbred, Michael B. Russell

**Affiliations:** ^1^Section of Medical Genetics, Department of Laboratory Medicine, Telemark Hospital, 3710 Skien, Norway; ^2^Head and Neck Research Group, Research Centre, Akershus University Hospital, Lørenskog, Norway; ^3^Campus Akershus University Hospital, University of Oslo, Nordbyhagen, Norway

## Abstract

Charcot-Marie-Tooth (CMT) disease is the most prevalent inherited neuropathy. Today more than 40 CMT genes have been identified. Diagnosing heterogeneous diseases by conventional Sanger sequencing is time consuming and expensive. Thus, more efficient and less costly methods are needed in clinical diagnostics. We included a population based sample of 81 CMT families. Gene mutations had previously been identified in 22 families; the remaining 59 families were analysed by next-generation sequencing. Thirty-two CMT genes and 19 genes causing other inherited neuropathies were included in a custom panel. Variants were classified into five pathogenicity classes by genotype-phenotype correlations and bioinformatics tools. Gene mutations, classified certainly or likely pathogenic, were identified in 37 (46%) of the 81 families. Point mutations in known CMT genes were identified in 21 families (26%), whereas four families (5%) had point mutations in other neuropathy genes, *ARHGEF10, POLG, SETX,* and *SOD1*. Eleven families (14%) carried the *PMP22* duplication and one family carried a *MPZ* duplication (1%). Most mutations were identified not only in known CMT genes but also in other neuropathy genes, emphasising that genetic analysis should not be restricted to CMT genes only. Next-generation sequencing is a cost-effective tool in diagnosis of CMT improving diagnostic precision and time efficiency.

## 1. Introduction

Charcot-Marie-Tooth (CMT) is the most common inherited neuropathy, affecting 40 to 81 cases per 100,000 in the Norwegian general population [1, 2]. CMT is clinically, neurophysiologically, and genetically heterogeneous. The clinical classification is based on age at onset, distribution of muscle weakness, sensory loss, walking difficulties, and foot deformities [3]. CMT is neurophysiologically subdivided into a demyelinating (CMT1) and axonal (CMT2) form depending on whether the median motor nerve conduction velocity (NCV) is below or above 38 m/s, respectively. A third form, intermediate CMT, has both demyelinating and axonal features and NCV between 25 and 45 m/s [2, 3].

The mode of inheritance is autosomal dominant, autosomal recessive, or X-linked [3]. At present more than 40 CMT genes have been identified and there are several genes associated with related conditions [4–8]. Genetic heterogeneity and pleiotropic genes, that is, mutations in different genes, cause a similar phenotype and mutations in a single gene cause different phenotypes, which adds to the complexity of CMT [3, 6, 7]. Furthermore, sporadic cases of CMT are not uncommon due to autosomal recessive inheritance, reduced penetrance, late onset, small family size, and* de novo* mutations [2, 8, 9].

The duplication of* PMP22* is the most common cause of CMT. The prevalence was ~15% in two Norwegian studies and up to 40% in other selected populations [2, 9–13]. Otherwise, CMT is caused by point-mutations, with rare exception of non-*PMP22 *copy-number variations (CNVs) [14, 15]. Establishing a genetic CMT diagnosis provides patients and families with information about prognosis and recurrence risk, as well as future options for specific treatment [16, 17].

Current strategy for diagnosing CMT is based on the clinical and neurophysiological phenotype. It is favourable to initially test CMT1 patients for the* PMP22* duplication due to its high prevalence. Genes are thereafter traditionally tested sequentially by Sanger sequencing, but the low prevalence of specific CMT point-mutations renders sequential testing unfavourable due to time and cost. Furthermore, most diagnostic laboratories only have capacity for sequencing a few genes [3, 7, 12, 17]. Hence, it is important to develop a more comprehensive approach for clinical diagnosis of heterogeneous disorders such as CMT, dystonia, hereditary spastic paraplegia (HSP), and Parkinson's disease [6]. Next-generation sequencing (NGS) makes it possible to sequence several genes in parallel and at a low cost compared to traditional methods.

We applied NGS on 59 CMT families from the Norwegian general population and sequenced 32 CMT genes along with 19 genes causing other inherited neuropathies, since the phenotypes of CMT, distal hereditary motor neuropathy (dHMN), and other inherited neuropathies overlap [3, 4, 6].

## 2. Materials and Methods

### 2.1. Study Population

People with CMT residing in eastern Akershus County, Norway, January 1, 1995, were included in the study [2]. Akershus County has rural and urban areas and was inhabited by 297, 539 persons [18]. A total of 245 affected persons from 116 CMT families were identified. DNA was available in 81 CMT families, 189 affected individuals. The neurophysiology among the families was 38 CMT1, 33 CMT2, two intermediate CMT, and 8 families with an unknown neurophysiological phenotype. The families were previously tested for the* PMP22 *duplication by real-time quantitative PCR and point mutations in* PMP22*,* GJB1*,* MPZ*,* LITAF*,* MFN2*, and* EGR2 *by conventional Sanger sequencing [2]. Later, a duplication of* MPZ* was identified in one CMT family, and another CMT family had a point mutation in the* SOD1* gene [14, 19]. A mutation was identified in 22 CMT families. A more comprehensive description of the study population has been published previously [2].

This study applied NGS on 70 affected individuals from 59 CMT families without a genetic diagnosis; these were 22 CMT1 families, 29 CMT2 families, one intermediate CMT family, and seven families with unknown neurophysiological phenotype. A control group of 180 healthy individuals were included in order to detect polymorphisms present in ≥1% of the population [20]. The Norwegian Regional Ethical Committee for Medical and Health Research approved the project, and the participants gave written informed consent.

### 2.2. Targeted Capture and DNA Sequencing

Table 1 shows the 51 neuropathy genes included in the panel [3, 4, 6]. Illumina's DesignStudio (Illumina Inc., San Diego, USA) for TruSeq Custom Enrichment was used to target all exons and flanking 5′ and 3′UTR (untranslated region) sequences (default settings). In total, 909 oligonucleotide probes covering 256,248 bp (base pairs) were included. Genomic DNA was extracted from whole blood using standard techniques; DNA samples were prepared in multiplex according to standard TruSeq Sample Prep and Custom Enrichment protocols (Illumina), and 75 base pairs were sequenced in each direction (paired-end). Sequencing was performed on the Illumina HiScan SQ. Samples from affected and controls were run in two separate runs.

### 2.3. Sequence Analysis

Bioinformatic analysis consisted of a standard protocol including image analysis and base calling by Illumina RTA 1.12.4.2, demultiplexing by CASAVA 1.8 (Illumina), and alignment of sequence reads to the reference genome GRCh37/hg19 by BWA [23]. Picard (http://picard.sourceforge.net/) was used for removing PCR duplicates. The GATK (Genome Analysis Toolkit) was applied for base quality score recalibration, INDEL (insertion and deletion) realignment, and SNP (single nucleotide polymorphism) and INDEL discovery [24, 25]. Annotation of sequence variants was performed by Annovar [26]. Variants present in exons ±10 bp intron sequences and 3′- and 5′UTR (untranslated region) were included in further analysis.

### 2.4. Classification of Variants

Variants were classified into five pathogenicity classes (Table 2). Variants were classified based on frequency data from 1000 genomes (http://www.1000genomes.org/), dbSNP 135 (http://www.ncbi.nlm.nih.gov/projects/SNP/), 180 in-house normal controls, pathogenicity predictions through the Alamut interface v2.2-0 (Interactive Biosoftware, Rouen, France), and reports in HGMD, IPNMDB, and the literature [4, 6, 27]. Variants with prevalence ≥ 0.1% in dbSNP 135 or 1000 genomes and presence in ≥ 2 in-house normal controls were removed unless homozygous or compound heterozygous. Variants with frequency < 0.1% were considered possible pathogenic as the SNP databases may contain information from individuals with disease, especially traits with debut during life such as CMT. Data from the ESP (the exome sequencing project) (http://evs.gs.washington.edu/EVS/) was also used in classification but only as a guidance as this database contains data from both the selected affected and controls for specific traits. Synonymous, intronic, and UTR variants not predicted to have an effect on splice site were also removed. The remaining variants were defined as the candidate variants. Variants classified likely or certainly pathogenic in recessive genes had to be present in a homozygous or compound heterozygous state. Classification into these classes also required phenotype-genotype correlation with previously published literature, and/or segregation of the variant(s) within the affected in the families, and/or the possible dual (digenic) effect of two variants in different genes. Identified variants classified certain, likely, and uncertain pathogenic were submitted to the ClinVar database (http://www.ncbi.nlm.nih.gov/clinvar/).

### 2.5. Verification by Sanger Sequencing

Candidate variants were verified by Sanger sequencing in all available family members to establish genotype-phenotype correlation. Primer design and sequence analysis were performed in CLC Main Workbench (CLC bio, Aarhus, Denmark); the sequencing was carried out using standard procedures and sequenced on the ABI3130XL (Life Technologies Ltd., Paisley, UK) as previously described [2].

## 3. Results

### 3.1. Sequencing Performance Results, Variant Identification, and Verification

Analysis of sequence data revealed uniform coverage and high read depths in all samples. On average among the affected patients, the percentage of nucleotides with at least 30x and 2x coverage was 97.73% and 98.73%, respectively, and the mean coverage depth was 516. On average, 202 variants were detected among the 51 investigated genes per patient. Table 1 shows sequence capture performance results per gene and Table 3 shows sequence capture performance and variant identification results among the 70 affected analyzed by NGS. In the group defined as candidate variants, 63 nonsynonymous exonic variants, zero synonymous variants, and four nonexonic variants remained among the 70 patients. The candidate variants were sorted in the five classes: (5) certainly pathogenic—seven variants, (4) likely pathogenic—ten variants, (3) uncertain pathogenic—15 variants, (2) unlikely pathogenic—15 variants, and (1) certainly not pathogenic—20 variants. All candidate variants were verified by Sanger sequencing.

### 3.2. Prevalence of CMT Variants

**Figure 1:**
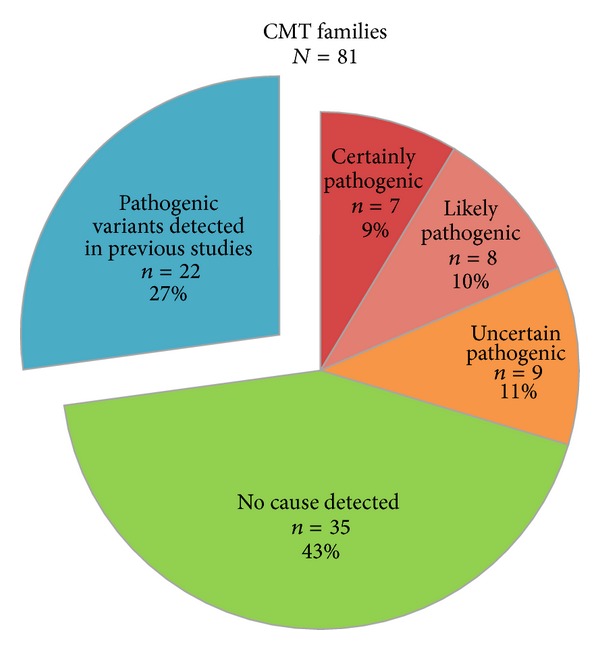
Identified variants in 81 Norwegian CMT families from the general population. Our previous studies identified copy-number variations in 12 CMT families and pathogenic point mutations in 10 CMT families [2, 14, 19]. The remaining 59 CMT families were investigated by next-generation sequencing.

The distribution of variants among the CMT families is illustrated in Figure 1.  Table 4 shows phenotype-genotype correlations for certain and likely pathogenic variants and Supplemental Table 1 (see Supplementary Material available online at http://dx.doi.org/10.1155/2014/210401) shows genotype-phenotype correlations for all candidate variants in the 59 CMT families analysed by NGS.

NGS identified seven certain, 10 likely, and 15 uncertain pathogenic variants in 24 CMT families. One family was compound heterozygote for likely pathogenic variants in* POLG* (family 62) and six CMT families had possible dual pathology, that is, mutations in two different genes. Family 252 had one certain variant and one uncertain variant in* SH3TC2* and* AARS*, respectively. Family 95 had two likely pathogenic variants in* REEP1* and* SETX*. Three families had one likely and one uncertain pathogenic variant,* LMNA* and* DCTN1* in family 27,* LM NA *and* ARHGEF10* in family 54, and* DYNC1H1* and* GAN *in family 231. Family 11 had two uncertain pathogenic variants in* SEPT9* and* SETX*. Eleven of the certain, likely, and uncertain pathogenic variants were novel. Two families had mutations in previously sequenced genes,* GJB1* in family 5 and* MFN2* in family 90. These were not detected in the previous study due to mix-up of DNA of an affected and unaffected and due to an unknown laboratory mistake.

**Figure 2:**
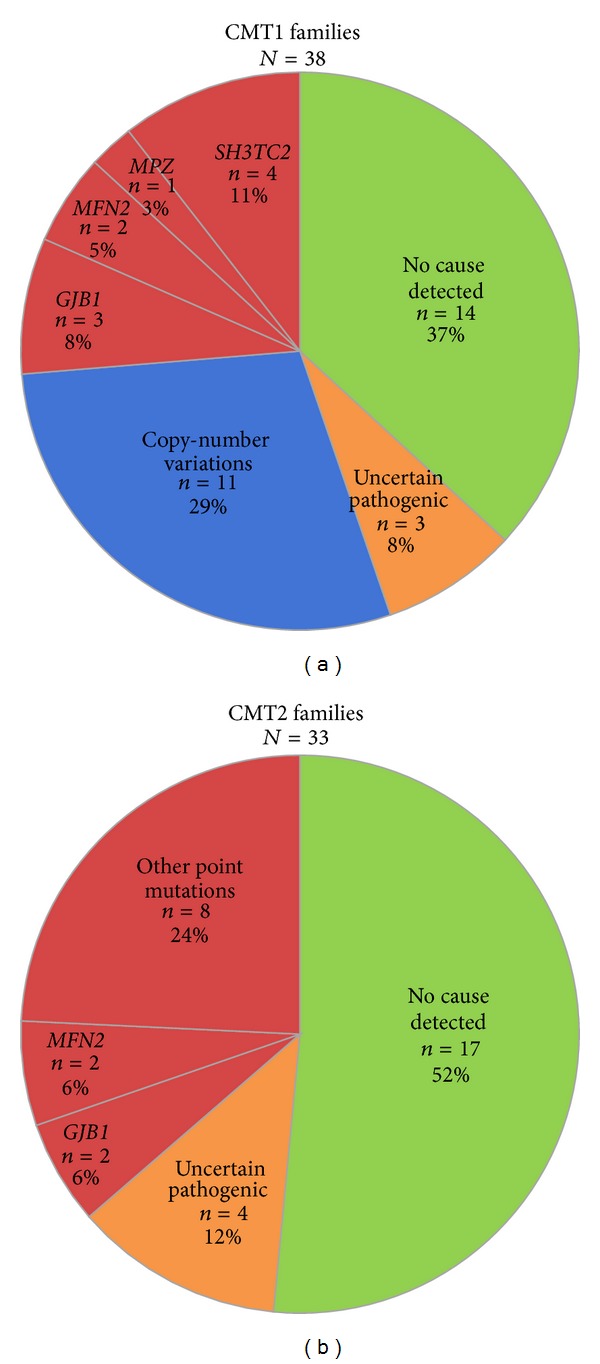
Frequencies of certain and likely pathogenic variants in CMT1 and CMT2 families from the Norwegian general population.

Of the total 81 CMT families, 37 CMT families had certain or likely pathogenic variants in 16 different genes (Table 4). Twelve CMT families had a CNV (11 families had the* PMP22* duplication and one family had a* MPZ* duplication) and 25 CMT families carried a point mutation.  Figure 2 illustrates the gene frequencies among the CMT1 and CMT2 subgroups. Of the 38 CMT1 families, 55% (21/38) of the families had certain or likely identified genotypes; that is, 29% (11/38) had a CNV and 26% (10/38) had a point mutation. Thirty-six percent (12/33) of the CMT2 families had a certain or likely identified genotype. One of the two families with intermediate CMT had an identified genotype. Among the eight families with unknown neurophysiological phenotype, one family had* PMP22* duplication and two families had point mutations. Four families had likely pathogenic variants in non-CMT genes,* ARHGEF10*,* POLG, REEP1*,* SETX, *and* SOD1* [4, 6]. Forty-one percent (11/27) of the sporadic case families had certain or likely identified genotypes; that is, three families had* PMP22* duplication and eight families had point mutations.

## 4. Discussion

### 4.1. Main Findings

This is to our knowledge the first study to provide prevalence data for most of the currently known CMT genes in a population based sample by targeted NGS. The main result of our study is as follows. After extracting CMT families with the* PMP22 *duplication, sequencing identifies certain and likely pathogenic point mutations in 36% (25/70) of the CMT families. The duplication of* PMP22* is the most common cause of CMT, found in 14% (11/81) of our families, whereas one family had a duplication of the* MPZ* [2, 14]. Large CNVs are not detected by our NGS-pipeline but require other methodologies, such as MLPA (multiplex ligation-dependent probe amplification). Thus, before NGS is applied, patients with CMT1 should be tested for the* PMP22 *duplication. Other CNVs are considered rare [48]. The known CMT genes accounted for the majority of our identified mutations supporting a correct clinical diagnosis. However, phenotypically certain CMT families had certain and likely pathogenic variants in the non-CMT neuropathy genes, that is,* ARHGEF10, POLG*,* SETX*, and* SOD1*, thus expanding the number of known CMT genes. This highlights the importance of including all neuropathy genes in the NGS panel due to the genetic heterogeneity and pleiotropic genes of inherited neuropathies.

### 4.2. Study Population

Our material included 27 CMT families with only one affected; that is, the diagnostic certainty of the phenotype might be less than in CMT families with several affected. However, it would be incorrect to exclude sporadic cases, as CMT may be caused by autosomal recessive inheritance, reduced penetrance, and* de novo* mutation as well as nonpaternity. Autosomal recessive CMT accounts for about 4% of all cases in Europe, while it is considerably more frequent in countries with a high rate of consanguinity [7].* De novo *duplication of* PMP22 *may occur in about 10% of the patients [8]. We identified the* PMP22 *duplication in three and a point mutation in eight of the total 27 sporadic CMT families. Thus, CMT variants were identified almost equally frequent in the sporadic and nonsporadic CMT families, that is,* PMP22* duplication 11% (3/27) versus 15% (8/54) and point mutations 30% (8/27) versus 35% (19/54), justifying the inclusion of the 27 sporadic CMT families in our material.

### 4.3. Methodological Considerations

Technically, the NGS panel demonstrated excellent results for coverage, read depth, and robustness for all genes in all 250 patients and controls, except one control with poor DNA quality. Lowering the possibility of technical errors is important in a clinical setting. NGS has several diagnostic advantages in heterogeneous diseases; that is, all known genes can be effectively sequenced and interpreted simultaneously. Furthermore, Sanger sequencing does not detect dual pathology, as sequencing is usually finalized when the first pathogenic variant is identified. This may be the reason why the literature rarely reports dual pathology, except from an American study which identified dual pathology in 1.4% of the CMT patients [9]. We identified possible dual pathology in 10% (6/59) of our CMT families. Thus, CMT dual pathology may not be as rare as the earlier literature implies. The digenic effect of two variants in different genes may modulate the phenotype, depending on whether the gene products work in the same pathways or not.

Another shortcoming with selective gene testing of a specific CMT phenotype is that unknown genotype-phenotype correlations are missed. An example is* MFN2, *usually tested only in CMT2 families; thus* MFN2 *mutations in CMT1 or intermediate CMT families are missed [49, 50].

The diagnostic benefit of NGS targeted sequencing has been highlighted in other heterogeneous diseases such as cardiomyopathies and epilepsy [51, 52]. The technical quality of NGS targeted sequencing has previously been questioned in relation to clinical diagnostics, but increasing quality is now obtained of which two examples are the study on cardiomyopathies and ours indicating that NGS targeted sequencing is ready for clinical diagnosis [52].

Exome sequencing is another NGS approach, where every exon in the genome is sequenced. It has been frequently applied on rare Mendelian disorders as well as on some CMT patients [42, 53]. Targeted sequencing as compared to exome sequencing shows higher technical performance, increased capacity per run (192 versus 12 samples in our laboratory), easier data analysis, lower cost of data storage, fewer problems with incidental findings, and lower cost (approximately € 175 (500x coverage) versus € 1165 (70x coverage) in our laboratory). Furthermore, it is easier to adopt in small laboratories. However, exome sequencing can discover new disease genes, while targeted sequencing only can if the gene panel is expanded. In families with unknown CMT genotype exome sequencing could be beneficial as a next step towards a genetic diagnose.

Precise classification of variants with exclusion of nonpathogenic and inclusion of pathogenic variants is extremely important in a clinical setting. Stringent criteria were applied, in order to avoid misclassification. The analysis of the 179 controls secured that ethnically specific normal variants were excluded. Detailed clinical data and family history were necessary for matching genetic data with the phenotype. A limitation with the interpretation of novel variants detected in this study is that no functional tests have been performed, but currently this is rarely available as part of routine genetic testing and beyond the scope of our present study.

### 4.4. Genotype-Phenotype Correlations

Among the seven certain pathogenic variants, four families were homozygous for the* SH3TC2 *Arg954* mutation, previously reported in several populations [6]. The prevalence of 5% (4/81) shows that* SH3TC2* should not be considered an unusual CMT gene in Northern Europe.

Eight CMT families had variants classified likely pathogenic. Phenotypically certain CMT families had pathogenic variants in the non-CMT neuropathy genes.* ARHGEF10* has been associated with slow NCV [39]. CMT2 and dHMN phenotypes have previously been reported for patients with* POLG* variants [21, 22].* SETX* variants are associated with dHMN among other phenotypes, but this patient had a neurophysiological CMT2 phenotype. In a previous study on the same material, the affected in a large CMT2 family carried a certain pathogenic variant in SOD1, a gene usually associated with ALS [19]. The identification of certain and likely pathogenic variants in non-CMT genes and in CMT genes, regarded unusual, is especially important in the CMT2 families, where an accurate molecular diagnosis often has been lacking. It could also be speculated whether these genes might be more common than first thought but has been considered unusual due to lack of routine analysis.

Among the 15 uncertain pathogenic variants, 9 families had heterozygous variants in* GAN*,* MTMR2*, and* SH3TC2* genes usually associated with autosomal recessive inheritance but dominant inheritance has also been reported in a few cases often related to lighter phenotypes [6]. In our cases the variants were predicted pathogenic and the phenotype matched previous reports for these genes, except for one variant in* MTMR2*. Further analysis is required in order to establish whether the heterozygous state can cause a mild phenotype.

The identification of dual pathology is important to increase our knowledge of interplay between different gene variants. Dual pathology was observed in six of our CMT families. One sporadic case with CMT2 and spasticity had likely pathogenic variants in* SETX* and* REEP1*; we assume the* SETX* variant causes CMT2 and the* REEP1* variant causes spasticity. Thus, in this case we do not consider* REEP1* a CMT causing gene. The pathology of the* LMNA* variant observed in two CMT families has been questioned due to extreme phenotypic diversity and low penetrance in affected families [44]. It is speculated that the pathogenic effect of this variant might be due to the digenic inheritance with another variant [44]; intriguingly this was observed in both our cases with variants in* ARHGEF10* and* DCTN1*.* LMNA* and* ARHGEF10* are both involved in myelination and cell morphology [39, 54].* LMNA* and* DCTN1* are situated in the same pathway, activation of the transcription factor* XBP1,* which has been associated with neuron differentiation [55].

### 4.5. Research in Context

**Figure 3:**
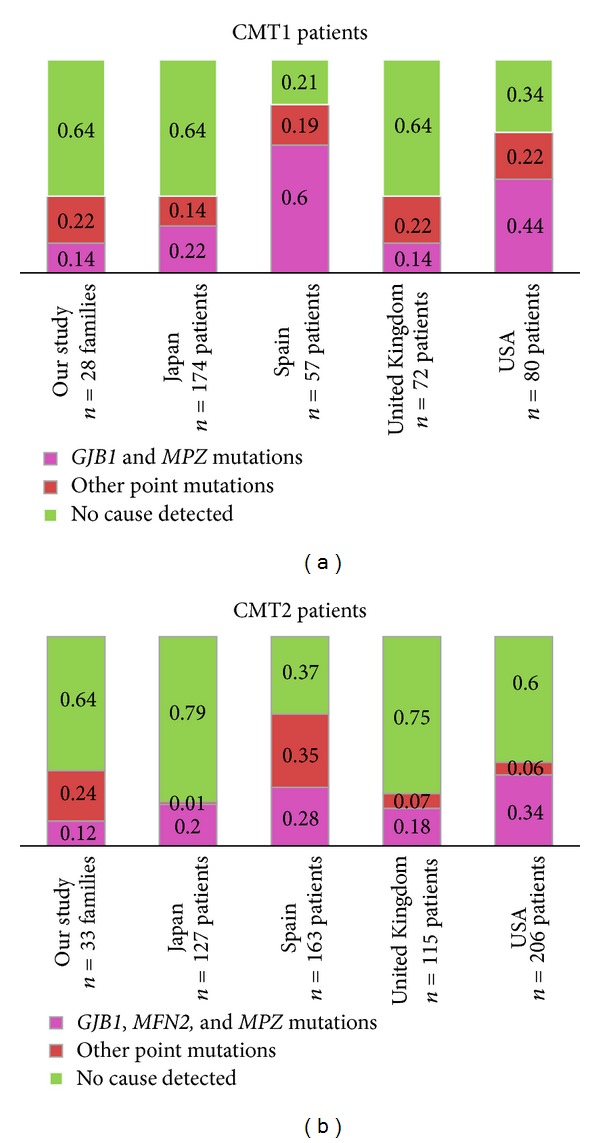
Frequencies of CMT1 and CMT2 point mutation in five studies [9, 10, 12, 13]. Numbers from our study include certainly and likely pathogenic variants, whereas the families with variants of uncertain pathogenicity have been included in the no cause detected group. Patients with intermediate forms of CMT were excluded in our study and the British and American studies but not in the Spanish and Japanese studies as intermediate forms were not differentiated; this may lead to a screw comparison. Numbers from the British study include only patients seen in their inherited neuropathy clinic and numbers from the American study include those with reported neurophysiology.

A comparison of our results with the prevalence of identified CMT point mutations in four large clinic populations of affected individuals from Japan, Spain, United Kingdom, and USA is shown in Figure 3 [9, 10, 12, 13].

After exclusion of the* PMP22* duplication, point mutations were detected in 36% of our, Japanese, and British CMT1 patients, 66% of the American CMT1 patients, and 79% of the Spanish CMT1 patients [9, 10, 12, 13]. Point mutations in* GJB1* or* MPZ*, the two genes most frequently mutated in CMT1,were identified in 44% of the American patients, 60% of the Spanish patients, and only 14–22% of the patients in the other three studies. We identified a higher percentage of pathogenic CMT2 variants than the British and Japanese studies but lower than the American and Spanish studies [9, 10, 12, 13].* GJB1*,* MPZ,* and* MFN2* variants, the most common causes of CMT2, accounted for 12% in our study and 18, 20, 28, and 34% in the British, Japanese, Spanish, and American studies. Apart from the* GJB1*,* MPZ,* and* MFN2* genes, variants were identified in 24% of our CMT2 families and in 35% of the Spanish patients but only in 1, 6, and 7% of the Japanese, American, and British CMT2 patients. High yield of identified “uncommon” CMT2 variants in our study and the Spanish study is most likely due to analysis of almost all CMT2 genes and the additional 19 neuropathy genes in our study. In the Spanish CMT2 patients, 26% had variants in* GDAP1, *also accounting for the high yield. In another study from northern Norway, CMT patients were analysed for the* PMP22* duplication and point mutations in seven genes (*EGR2*,* GJB1*,* LITAF*,* MPZ*,* MFN2*,* NEFL*, and* PMP22*), a genetic diagnose was established in only 17% of the patients [11]. These results together with ours indicate that other genotypes might be more common in Norway. At least part of the gene frequency differences is likely due to geographical differences, while different ascertainment might also affect the results. This emphasizes the difficulties of having a common sequential testing scheme for a rationale diagnosis of CMT but rather highlights the benefits of NGS targeted analysis.

Why do half of the CMT families lack a molecular diagnosis in our study? Several CMT genes are still to be identified, and there might be unidentified founder variants in the Norwegian population. After the analysis of these families, more than ten new genes have rapidly been identified mainly due to exome sequencing; these may count for a few unidentified cases [5, 6, 8]. Small tandem repeats, copy-number variations, mutations in regulatory elements distant from the gene, or cellular changes other than mutations in genomic DNA might be relevant in some cases. Dual pathology is easy to overlook. We also applied stringent criteria for the classification of variants; all heterozygous variants in autosomal recessive genes were classified as uncertain or unlikely pathogenic and variants with prevalence ≥ 0.1% in 1000 genomes or dbSNP135 were removed; thus some of these might be pathogenic. Clinical misclassification cannot be ruled out but it probably explains only a minority of the cases, since pathogenic variants were identified equally frequent in familial and sporadic cases.

## 5. Conclusion

Sequential testing scheme is useful for the* PMP22 *duplication as an initial first step in CMT1; otherwise it is advantageous to start with NGS targeted sequencing.

The insight of pathological mechanisms caused by mutations in CMT genes has prompted promising reports of specific targeted treatments. Examples are treatment with HDAC6 inhibitors in* HSPB1* mutant mice, restoring axonal transport defects [16], and treatment with curcumin improving outcome of neuropathy in* MPZ* mutant mice [56]. Specific treatments require a precise genetic diagnose. The NGS technology has now become a robust and powerful tool with high technical quality, delivering increased diagnostic precision at a low cost. The NGS technology is likely to change clinical practice in complex diseases over the next years.

## Supplementary Material

Supplementary table 1: provides clinical characterization of the 59 CMT families analysed by NGS and a description of the candidate variants identified. The definition of a candidate genetic variant is given in materials and methods.

## Figures and Tables

**Table 1:** Sequence capture performance results for the 51 neuropathy genes included in the panel.

Gene	GenBank accession and version number	Neuropathy phenotype	Average coverage	% Bases ≥ 2x coverage	% Bases ≥ 30x coverage
*AARS *	NM_001605.2	CMT2	642	100.0	100.0
*ARHGEF10 *	NM_014629.2	Slow NCV	620	100.0	97.7
*ATP7A *	NM_000052.6	dHMN	295	100.0	94.4
*BSCL2 *	NM_001122955.3	dHMN	845	100.0	100.0
*CTDP1 *	NM_004715.4	CCFDN	488	100.0	87.7
*DCTN1 *	NM_001135041.2	dHMN	972	100.0	99.8
*DNM2 *	NM_001005361.2	CMT2 and ICMT	449	100.0	96.6
*DYNC1H1 *	NM_001376.4	CMT2	662	100.0	99.3
*EGR2 *	NM_000399.3	CMT1, DSN, and CMT4	1328	100.0	100.0
*FAM134B *	NM_001034850.2	HSAN	296	100.0	88.5
*FGD4 *	NM_139241.2	CMT4	410	100.0	100.0
*FIG4 *	NM_014845.5	CMT4	480	100.0	100.0
*GAN *	NM_022041.3	GAN	472	100.0	93.1
*GARS *	NM_002047.2	CMT2, dHMN	427	100.0	92.9
*GDAP1 *	NM_001040875.2	CMT2, CMT4, and ICMT	591	100.0	100.0
*GJB1 *	NM_000166.5	CMTX	513	100.0	98.0
*HK1 *	NM_033500.2	CMT4	654	100.0	95.9
*HSPB1 *	NM_001540.3	CMT2 and dHMN	429	100.0	80.4
*HSPB3 *	NM_006308.2	dHMN	535	100.0	100.0
*HSPB8 *	NM_014365.2	CMT2 and dHMN	536	100.0	96.9
*IGHMBP2 *	NM_002180.2	dHMN	540	100.0	99.2
*IKBKAP *	NM_003640.3	HSAN	488	100.0	99.8
*KIF1B *	NM_015074.3	CMT2	579	99.9	98.9
*LITAF *	NM_001136472.1	CMT1	454	100.0	90.0
*LMNA *	NM_170708.3	CMT2	533	100.0	93.8
*MED25 *	NM_030973.3	CMT2	476	100.0	84.4
*MFN2 *	NM_001127660.1	CMT2	602	100.0	99.3
*MPZ *	NM_000530.6	CMT1, CMT2, ICMT, and DSN	417	100.0	82.2
*MTMR2 *	NM_016156.5	CMT4	338	100.0	97.5
*NDRG1 *	NM_001135242.1	CMT4	501	100.0	97.2
*NEFL *	NM_006158.4	CMT1 and CMT2	439	100.0	98.5
*NGF *	NM_002506.2	HSAN	475	100.0	87.8
*NTRK1 *	NM_001012331.1	HSAN	528	100.0	85.8
*PLEKHG5 *	NM_001042664.1	ICMT and dHMN	463	99.5	95.2
*PMP22 *	NM_153322.2	CMT1, DSN, and HNPP	597	100.0	100.0
*POLG *	NM_001126131.1	CMT associated [21, 22]	485	100.0	95.5
*PRPS1 *	NM_002764.3	CMTX	373	100.0	99.0
*PRX *	NM_181882.2	CMT4 and DSN	966	100.0	100.0
*RAB7 *	NM_004637.5	CMT2	486	100.0	98.4
*REEP1 *	NM_001164731.1	dHMN	476	100.0	95.5
*SBF2 *	NM_030962.3	CMT4	443	100.0	97.4
*SEPT9 *	NM_001113493.1	HNA	449	100.0	86.3
*SETX *	NM_015046.5	dHMN	566	100.0	99.4
*SH3TC2 *	NM_024577.3	CMT4	506	100.0	100.0
*SLC12A6 *	NM_001042497.1	ACCPN	760	100.0	99.3
*SOD1 *	NM_000454.4	CMT associated [19]	485	100.0	100.0
*SOX10 *	NM_006941.3	PCWH	311	98.8	77.5
*SPTLC1 *	NM_001281303.1	HSAN	449	100.0	95.7
*TRPV4 *	NM_001177428.1	CMT2 and dHMN	454	100.0	100.0
*WNK1 *	NM_014823.2	HSAN	883	100.0	99.1
*YARS *	NM_003680.3	ICMT	475	100.0	95.0

ACCPN = agenesis of the corpus callosum with peripheral neuropathy; CCFDN = cataract, congenital, with facial dysmorphism and neuropathy; CMT1 = demyelinating Charcot-Marie-Tooth disease with autosomal dominant inheritance; CMT2 = axonal Charcot-Marie-Tooth disease; CMT4 = demyelinating Charcot-Marie-Tooth disease with autosomal recessive inheritance; CMTX = Charcot-Marie-Tooth disease, X-linked inheritance; dHMN = distal hereditary motor neuronopathy; DSN = Dejerine-Sottas neuropathy; *GAN* = giant axonal neuropathy; HNPP = hereditary neuropathy with liability to pressure palsies; HSAN = hereditary sensory and autonomic neuropathies; ICMT = intermediate Charcot-Marie-Tooth disease; NCV = nerve conduction velocity; PCWH = peripheral demyelinating neuropathy, central dysmyelination.

**Table 2:** Classification of variants into five pathogenicity classes.

Pathogenicity class	Conclusion	Criteria
5	certainly pathogenic	(1) Reported pathogenic in at least two unrelated cases (2) and/or functional studies reveal effect on protein structure/function (3) and zygosity/inheritance of phenotype fits the variant (4) and phenotype-genotype correlation with previously published literature

4	likely pathogenic	(1) Reported pathogenic in one case (2) and/or predicted pathogenic in at least 2 of 4 variant prediction tools: SIFT [28], Polyphen [29], Align GVGD [30], and Mutation Taster [31] through the Alamut interface (3) and/or predicted loss or gain of splice site predicted in at least 4 of 5 splice site predictors: SpliceSiteFinder [32], MaxEntScan [33], NNSPLICE [34], GeneSplicer [35], and Human Splicing Finder [36] through the Alamut interface (4) and/or close proximity to known pathogenic mutations with similar or lower variant prediction score (5) and zygosity/inheritance of phenotype fits the variant (6) and phenotype-genotype correlation with previously published literature

3	uncertain pathogenic	(1) Present in ≤0.1% of dbSNP135 or 1000 genomes (2) and/or present in ≤1 in-house control (3) and zygosity/inheritance of phenotype in family fits the variant (4) Variants in class 2 may be lifted to this class if present in several affected patients with similar phenotype

2	unlikely pathogenic	(1) Present in 0.1–1% of dbSNP135 or 1000 genomes (2) and/or present in 2-3 in-house controls (3) and/or predicted no loss or gain of splice site predicted by 5/5 splice site predictors (applies only to synonymous variants and variants in introns and UTRs) (4) and/or reported benign in the literature

1	certainly not pathogenic	(1) Present in ≥1% of dbSNP135 or 1000 genomes (2) and/or present in ≥4 in-house controls

dbSNP = the single nucleotide polymorphism database.

**Table 3:** Sequence capture performance results and variant identification among 70 affected patients.

		Average	Standard deviation	Min	Max
Coverage	Coverage, all regions^1^	515.5	105.1	77.1	828.7
	% Base ≥ 2x coverage, all regions^1^	99.0	0.001	98.6	100.0
	% Base ≥ 30x coverage, all regions^1^	97.7	0.007	93.5	99.2

Variant identification	Variants in all regions^1^	202	18.2	163	241
	Variants in all regions^1^ after filtering^2^	11	3.2	6	21
	Nonsynonymous variants in exons^2^	3	1.4	1	7
	Synonymous variants in exons^2^	3	1.0	2	6
	Variants in ± 10 bp intron, 3′UTR and 5′UTR variants^2^	5	2.7	2	14

^1^All regions = exons ± 10 bp intron sequence, 3′UTR and 5′UTR.

^2^Filtering against presence in ≥1% of dbSNP135 or 1000 genomes and presence in ≥4 in-house unrelated controls.

bp = base pair; UTR = untranslated region.

**Table 4:** The genotype-phenotype correlation in 81 Norwegian CMT families carrying certain or likely pathogenic variants.

Gene^1^	Nucleotide change	Protein change	Family ID	CMT type	Genotype-phenotype correlation
Certainly pathogenic					
*GJB1 *	c.688C>T	p.Arg230Cys	5	CMT2	Family with axonal CMT and X-linked inheritance. Previously reported in [37].
*HSPB1 *	c.380G>T	p.Arg127Leu	102	CMT2	Novel variant, highly conserved, predicted pathogenic. Classified certainly pathogenic as previously reported CMT families had a pathogenic variant in the same codon, causing p.Arg127Trp [6]. Present in an affected patient and his affected daughter, not in his unaffected daughter.
*MFN2 *	c.310C>T	p.Arg104Trp	90	CMT1	Severely affected CMT1 patient with slightly decreased motor NCV, 36 m/s. Previously reported to cause early onset severe CMT2 by several [38].
*SH3TC2 *	c.2860C>T	p.Arg954^∗2^	142, 252, 285, 295	CMT1	Present as homozygous in four patients from four different families with demyelinating CMT. Reported to cause CMT1 in several populations [6]. Scoliosis at variable degree was found in all cases, which is often associated with mutations in this gene. Found as heterozygous in ten unaffected family members and in five in-house controls.

Likely pathogenic					
*ARHGEF10 *	c.1013G>C	p.Arg338Thr	257	CMT2	Novel variant, highly conserved, predicted benign but extensive change in amino acid physiochemical properties. Sporadic case with CMT2 and decreased NCV. Close proximity to another heterozygous variant (Thr332Ile) associated with decreased NCV and thin myelination [39]. Functional studies show that the Thr332Ile mutant stimulates increased actomyosin contraction, regulating cell morphology in Schwann cells [40]. Classified likely pathogenic due to similar phenotype, NCV in the same range, and close proximity to the previous reported variant.
*DNM2 *	c.1241A>G	p.Lys414Arg	9	CMT	Totally conserved, predicted pathogenic, situated in the dynamitin central domain. Sporadic case with unknown CMT. Not present in the unaffected daughter but in one in-house control and in one control in the ESP database; but considering the relatively high age of onset (85 years), it is uncertain whether these controls could develop neuropathy at higher age or whether the variant display reduced penetrance. Variants in* DNM2* cause both axonal CMT and intermediate CMT. Situated in the same domain as another variant associated with CMT [41].
*DYNC1H1 *	c.1700G>A	p.Arg567His	231	CMT2	Novel variant, highly conserved situated in the dynein heavy chain, domain-1. Recently discovered as a CMT causing gene, reported to cause autosomal dominant CMT [42]. The previously reported variant (His306Arg) is situated in the same highly conserved domain as our variant and apart from some higher age of onset in our family the phenotypes correlate well. DNA was only available from one case in this family.
*KIF1B *	c.881A>G	p.Lys294Arg	123	CMT2	Totally conserved, predicted pathogenic, situated in the kinesin motor domain, found among one individual in the ESP database (0.008%). Situated in the same highly conserved domain as a heterozygous variant (Gln98Leu) reported in another CMT2 family [43]. Researchers have been cautious about classifying *KIF1B* a CMT causing gene since only one family has been reported. As functional studies of the previously reported variant have confirmed loss of motor activity and variants in other motor proteins (*KIF1A*, *DYNC1H1*, and *DCTN1)* also are involved in neuropathy, we consider *KIF1B* a possible CMT causing gene and classify our variant likely pathogenic. DNA was only available from one case in this family.
*LMNA *	c.1930C>T	p.Arg644Cys	27 54	CMT CMT2	Totally conserved, predicted pathogenic. The pathogenicity of this variant has been questioned due to extreme phenotypic diversity including neuropathy and also low penetrance in affected families. But in support of its pathogenicity, found in 19 patients and not in 1000 controls (including our results), totally conserved, and studies of fibroblast carrying this variant show abnormalities of nuclear shape [44]. Found in the ESP database among 14 individuals (0.1%), but included in this database are also the affected carrying traits associated with *LMNA* mutations. Digenic inheritance with another variant, which is observed in three reported cases, may explain the phenotypic diversity and nonpenetrance [44]. Intriguingly, the CMT family with unknown neurophysiology carried a heterozygous variant in *DCTN1*, p.Arg651Trp and the sporadic CMT2 case carried a heterozygous variant in *ARHGEF10*, p.His733Tyr, both classified uncertain pathogenic.
*POLG *	c.1491G>C c.2243G>C	p.Gln497His p.Trp748Ser	62	CMT2	p.Gln49His: highly conserved, predicted pathogenic. p.Trp748Ser: highly conserved, predicted pathogenic in one tool. Situated in the same domain. Sporadic case with CMT2. These two variants are reported to cause severe ataxic neuropathy with additional features in two Norwegian patients as compound homozygous [45]. Additionally the p.Trp748Ser variant is reported to cause neurodegenerative disorders with ataxia in three patients and dHMN in five patients as compound heterozygote [22, 46]. A patient in our clinic with simular phenotype presented with the same two variants, not seen in in-house controls or in SNP databases.
*REEP1* *SETX *	c.524A>G c.3075_3076insTGA	p.∗175Trpext∗55 p.Arg1026∗	95	CMT2	REEP1: novel stop loss variant lengthening the protein by 55 residues. SETX: novel nonsense (stop codon) insertion at position 1026, shortening the protein by 1652 amino acids. Sporadic case with CMT2 and spasticity. Variants in *SETX* are associated with dHMN, a phenotype that overlaps with CMT2 and variants in REEP1 are associated with hereditary spastic paraplegia and in some cases dHMN [6, 47]. Thus, we assume digenic pathogenicity: the *SETX* variant causing CMT2 and the *REEP1* variant causing spasticity.

Pathogenic variants identified among the same epidemiological population, prior to the NGS study [2, 14, 19]					
*GJB1 *	c.187G>A	Val63Ile	118, 256	CMT1	
*GJB1 *	225delG	Leu76Cysfs∗8	83	ICMT	
*GJB1 *	c.491G>A	Arg164Gln	44	CMT1	
*GJB1 *	c.658C>T	Arg220∗	398	CMT2	
*MFN2 *	c.280C>T	Arg94Trp	8	CMT1	
*MFN2 *	c.281G>A	Arg94Gln	38	CMT2	
*MFN2 *	c.2119C>T	Arg707Trp	258	CMT2	
*MPZ *	c.161C>G	Ser54Cys	39	CMT1	
*MPZ *	Duplication		124	CMT1	
*PMP22 *	Duplication		17, 51 56, 82, 136, 148, 155, 225, 309, 367	CMT1	
*PMP22 *	Duplication		371	CMT	
*SOD1 *	c.140A>G	His46Arg	1	CMT2	

^1^All relevant GenBank accession and version numbers are given in Table 1.

^2^Present as homozygous.

CMT = unspecified Charcot-Marie-Tooth; CMT1 = demyelinating Charcot-Marie-Tooth; CMT2 = axonal Charcot-Marie-Tooth; dHMN = distal hereditary neuronopathy; ESP = the exome sequencing project; ICMT = intermediate Charcot-Marie-Tooth; NCV = nerve conduction velocity.
