# Supplementary material for: Genetic Diagnosis of Charcot-Marie-Tooth Disease in a Population by Next-Generation Sequencing
Source: Biomed Res Int. 2014 Jun 16;2014:210401. doi: 10.1155/2014/210401 (PMC4082881; doi:10.1155/2014/210401)
Supplement: Supplementary file 1 — Supplementary table 1: provides clinical characterization of the 59 CMT families analysed by NGS and a description of the candidate variants identified. The definition of a candidate genetic variant is given in materials and methods. [file 210401.f1.pdf]

SUPPLEMENTAL TABLE 1:

Clinical characterization of patients in 59 families with CMT, candidate genetic variants and interpretation

<table>
<tr><th></th><th colspan="4">Clinical characterization</th><th colspan="5">Genetic results</th><th colspan="5">Validation and classification</th></tr>
<tr><th>Family (patient)[1]</th><th>Sex</th><th>Age at onset (years)</th><th>CMT type</th><th>Inheritance in family</th><th>Gene</th><th>Nucleotide change</th><th>Protein change</th><th>GenBank Accession number</th><th>Haplo -type</th><th>Present in-house controls</th><th>Present 1000g/ ESP[2] (%)</th><th>dbSNP number</th><th>Ranked pathogenic in VPD[3]</th><th>Class</th></tr>
<tr><td rowspan="3">5 (75)</td><td rowspan="2">M</td><td rowspan="2">38</td><td rowspan="2">CMT2</td><td rowspan="2">XL</td><td>GJB1</td><td>c.688C>T</td><td>p.R230C</td><td>NM_000166.5</td><td>hemi</td><td>-</td><td>-/-</td><td>-</td><td>2/4</td><td>5</td></tr>
<tr><td>WNK1</td><td>c.2218_2219insT</td><td>p.L740fs</td><td>NM_014823.2</td><td>het</td><td>-</td><td>-/-</td><td>-</td><td>n.a.</td><td>1</td></tr>
<tr><td colspan="14">GJB1 p.R230C

Interpretation: Variants in GJB1 are associated with both axonal and demyelinating CMT, heterozygous females are unaffected or more mildly affected than hemizygous males. This variant is previous reported pathogenic and confirmed pathogenic in cellular studies [1,2]. The variant presented as hemizygous in this patient and the patients affected brother (patient 77). By Sanger sequencing the variant was found as heterozygous in the unaffected daughter of patient 77 and not found in 13 other unaffected family members. The variant was not identified in the previous study by Braathen and colleagues [3] due to genetic analysis of patient 76 which later was diagnosed with symptomatic neuropathy. Genotype correlates with phenotype in the family.

Classification: Certainly pathogenic

WNK1 p.L740fs

Interpretation: Variants in this gene are associated with sensory and autonomic neuropathy AR type, onset in 1st decade, slow progression, decreased sensory NCV, impaired sensation and taste, ulcers and gastrointestinal reflux. No correlation with the patient’s phenotype and no segregating with the family phenotype as four unaffected family members carried the variant.

Classification: Certainly not pathogenic</td></tr>
<tr><td rowspan="2">5 (76)</td><td>M</td><td>-</td><td>-</td><td>XL</td><td>WNK1</td><td>c.2218_2219insT</td><td>p.L740fs</td><td>NM_014823.2</td><td>het</td><td>-</td><td>-/-</td><td>-</td><td>n.a.</td><td>1</td></tr>
<tr><td colspan="14">Interpretation: See patient 75 for interpretation of this variant. By closer examination it was also discovered that this patient had a symptomatic neuropathy.

Classification: Certainly not pathogenic</td></tr>
<tr><td rowspan="2">5 (77)</td><td>M</td><td>59</td><td>CMT2</td><td>XL</td><td>GJB1</td><td>c.688C>T</td><td>p.R230C</td><td>NM_000166.5</td><td>hemi</td><td>-</td><td>-/-</td><td>-</td><td>2/4</td><td>5</td></tr>
<tr><td colspan="14">Interpretation: See patient 75 for interpretation of this variant.

Classification: Certainly pathogenic</td></tr>
<tr><td rowspan="2">6 (87)</td><td rowspan="2">M</td><td rowspan="2">57</td><td rowspan="2">CMT2</td><td rowspan="2">AD</td><td>SH3TC2</td><td>c.3686A>T</td><td>p.D1229V</td><td>NM_024577.3</td><td>het</td><td>1</td><td>0.32/0.35</td><td>rs146920285</td><td>4/4</td><td>1</td></tr>
<tr><td>GJB1</td><td>c.-6G>A</td><td>-</td><td>NM_000166.5</td><td>hemi</td><td>-</td><td>-/0.03</td><td>rs201344743</td><td>n.a.</td><td>1</td></tr>
</table>

	SH3TC2 p.D1229V<br><br>Interpretation: Variants in this gene are associated with CMT1 AR form, variable onset and phenotype. This variant is previously reported possible pathogenic as heterozygous in two patients [4], but is also present in 1000g at more than 0.1% and in one in-house control. This patient had CMT2, two unaffected family members carried the variant whereas three affected family members did not. No genotype-phenotype correlation.<br><br>Classification: Certainly not pathogenic<br><br>*GJB1* c.-6G>A<br><br>Interpretation: Variants in *GJB1* are associated with both axonal and demyelinating CMT. This variant was predicted to have a small effect on splice site. [footnote 4] The genotype correlates well with the phenotype of this patient, but the variant was not seen in three other affected family members. No genotype-phenotype correlation.<br><br>Classification: Certainly not pathogenic													
6 (93)	M	82	CMT2	AD	*PRX*	c.823C>A<br>c.2612T>C	p.L275I<br>p.V871A	NM_181882.2	comp. het	-<br>141	-/0.07<br>-/-	rs200033507<br>-	2/4<br>0/4	1
	Interpretation: Variants in this gene are associated with DSN AR type. This patient has a CMT2 phenotype and the variants were not present as compound heterozygous in other affected family members. The p.L275I variant was also seen in family 78 where it neither segregated with the family phenotype.<br><br>Classification: Certainly not pathogenic													
7 (79)	M	61	CMT1	D	*IKBKAP*	c.3213G>C	p.E1071D	NM_003640.3	het	-	-/0.06	rs140024352	0/4	1
					SBF2	c.3292C>G<br>c.4955A>G	p.L1098V<br>p.K1652R	NM_030962.3	comp. het	10<br>1	1.00/2.12<br>-/-	rs117957652<br>-	1/4<br>2/4	2
					SH3TC2	c.1298C>T	p.S433L	NM_024577.3	het	2	-/0.07	-	3/4	1
	IKBKAP p.E1071D<br><br>Interpretation: Variants in this gene are associated with sensory and autonomic neuropathy AR form, onset at birth, progressive, predominant autonomic with eyes, mouth, vascular, gastrointestinal, kidney and skeletal involvement as well as decreased pain and temperature perception. This heterozygous variant was predicted benign, situated in a minor conserved area, and not in close proximity to other pathogenic variants. No phenotype correlation with previous reported literature and later age of onset in our patient.<br><br>Classification: Certainly not pathogenic<br><br>*SBF2* p.L1098V + p.K1652R<br><br>Interpretation: Variants in this gene are associated with CMT1 AR type, onset in 1st or 2nd decade, severe, abnormal myelin folding, severe distal sensory impairment and glaucoma. The two													

<table>
<tr><td></td><td colspan="14">variants identified in this patient are both predicted pathogenic in one and two VPD respectively. The p.L1098V variant is relatively common, whereas the p.K1652R variant is only observed in one in-house control. The inheritance pattern of this patient is however dominant not recessive and age of onset is later. It could be debated whether the p.K1652R variant might cause disease as heterozygous as it was only present in one in-house control and not seen in SNP databases, we consider this however unlikely as no disease causing heterozygous variants have been reported for this gene, the variant is situated in minor conserved area and the variant creates a small change in amino acid physiochemical properties.

Classification: Unlikely pathogenic

SH3TC2 p. S433L

Interpretation: Variants in this gene are associated with CMT1 AR type, variable onset and phenotype. This variant is predicted pathogenic in three VPD, but it was also present in two in-house controls and the ESP database at 0.07%. The patient’s likely unaffected child carries the variant and also another patient (family 45) with another CMT phenotype (CMT2) and her unaffected father.

Classification: Certainly not pathogenic

SBF2 and SH3TC2 are not connected in the same molecular pathways or processes.[footnote 5]</td></tr>
<tr><td rowspan="2">9 (80)</td><td>F</td><td>85</td><td>CMT</td><td>Sporadic</td><td>DNM2</td><td>c.1241A>G</td><td>p.K414R</td><td>NM_001005361.2</td><td>het</td><td>1</td><td>-/0.01</td><td>rs199927590</td><td>3/4</td><td>4</td></tr>
<tr><td colspan="14">Interpretation: Variants in this gene are associated with DI-CMT AD form, onset in 1st or 2nd decade. Totally conserved variant, predicted pathogenic, situated in the dynamitin central domain. Not present in the unaffected daughter. Situated in the same domain as another variant associated with CMT [5]. Apart from higher age of onset in our case, the phenotype correlates well with previous published literature. The variant was however present in one in-house control and among one control in the ESP database, but considering the relatively high age of onset, it is uncertain whether these controls could develop neuropathy at higher age or whether the variant show reduced penetrance.

Classification: Likely pathogenic</td></tr>
<tr><td rowspan="3">10 (92)</td><td rowspan="2">M</td><td rowspan="2">54</td><td rowspan="2">CMT1</td><td rowspan="2">Sporadic</td><td>IGHMBP2</td><td>c.547C>T</td><td>p.H183Y</td><td>NM_002180.2</td><td>het</td><td>1</td><td>-/-</td><td>-</td><td>0/4</td><td>1</td></tr>
<tr><td>SETX</td><td>c.7640T>C</td><td>p.I2547T</td><td>NM_015046.5</td><td>het</td><td>-</td><td>-/0.54</td><td>rs1511117904</td><td>0/4</td><td>2</td></tr>
<tr><td colspan="14">IGHMBP2 p.H183Y

Interpretation: Variants in this gene are associated with dHMN AR type, onset within first 3 months, death within 12 months, low birth weight and failure to thrive, hypotonia, diaphragmatic paralysis and severe respiratory failure. This heterozygous variant is predicted benign and is not conserved. Reported phenotype does not correlate with patient’s phenotype.

Classification: Certainly not pathogenic

SETX p.I2547T

Interpretation: Heterozygous variants in SETX are associated with ALS, dHMN, and tremor-ataxia syndrome. Homozygous or compound heterozygous variants have been associated with AOA2 and spinocerebellar ataxia. This variant has been reported pathogenic as heterozygous in one patient with tremor, ataxia and mild motor neuron signs [6]. But this variant is also relatively common and present in the ESP at 0.5% and in another patient with another CMT phenotype (CMT2, family 257).</td></tr>
</table>

<table>
<tr><td></td><td colspan="14">Classification: Unlikely pathogenic

IGHMBP2 and *SETX* are not connected in the same molecular pathways or processes.[footnote 5]</td></tr>
<tr><td rowspan="4">11 (140)</td><td rowspan="3">M</td><td rowspan="3">52</td><td rowspan="3">CMT1</td><td rowspan="3">Sporadic</td><td>*SEPT9*</td><td>c.530G>A</td><td>p.R177H</td><td>NM_001113495.1</td><td>het</td><td>-</td><td>-/-</td><td>-</td><td>3/4</td><td>3</td></tr>
<tr><td>*ATP7A*</td><td>c.3449T>C</td><td>p.I1150T</td><td>NM_000052.6</td><td>hemi</td><td>-</td><td>-/0.03</td><td>-</td><td>1/4</td><td>2</td></tr>
<tr><td>*SETX*</td><td>c.6792A>G</td><td>p.I2264M</td><td>NM_015046.5</td><td>het</td><td>-</td><td>-/0.02</td><td>rs148041889</td><td>2/4</td><td>3</td></tr>
<tr><td colspan="14">*SEPT9* p.R177H

Interpretation: Variants in this gene is associated with HNA AD form, onset in 1st to 3rd decade, episodes of pain followed by weakness and atrophy, neuropathy and facial dysmorphic features. Variants in this gene are also associated with cancer. The variant is predicted pathogenic in three VPD and situated in a well conserved area, but the genotype does not correlate with the phenotype of this patient.

Classification: Uncertain pathogenic

ATP7A p.I1150T

Interpretation: Some variants in this gene are associated with dHMN XD form (DSMAX), onset usually in 1st decade, slow progression. Most variants in this gene are however associated with other phenotypes like Menkes syndrome and occipital horn syndrome. This hemizygous variant gives a large amino acid substitution from a hydrophobic to polar uncharged amino acid, and is well conserved. The variant is situated in close proximity to variants associated with Menkes syndrome and occipital horn syndrome. The phenotype of this patient correlates however not with reported phenotypes for this gene.

Classification: Unlikely pathogenic

SETX p.I2264M

Interpretation: Heterozygous variants in *SETX* are associated with ALS, dHMN, and tremor-ataxia syndrome. Homozygous or compound heterozygous variants have been associated with AOA2 and spinocerebellar ataxia. This variant has been associated with AOA2 in two Norwegian individuals as homozygous and compound heterozygous [7]. This variant might give rise to peripheral neuropathy in a heterozygous state as dHMN is part of the clinical picture in AOA2 and also as this variant was predicted pathogenic in two VPD, totally conserved and only reported in two individuals in the ESP database. The phenotype of this patient is however CMT1.

Classification: Uncertain pathogenic

SEPT9, ATP7A and/or *SETX* are not connected in the same molecular pathway or processes.[footnote 5]</td></tr>
<tr><td rowspan="2">12 (184)</td><td rowspan="2">F</td><td rowspan="2">51</td><td rowspan="2">CMT</td><td rowspan="2">D</td><td>*SEPT9*</td><td>c.85C>A</td><td>p.P29T</td><td>NM_001113493.1</td><td>het</td><td>-</td><td>-/0.1</td><td>-</td><td>1/4</td><td>2</td></tr>
<tr><td>*PRX*</td><td>c.485A>C
c.2612T>C</td><td>p.K162T
p.V871A</td><td>NM_181882.2</td><td>comp. het</td><td>-
141</td><td>-/-
-/-</td><td>-
rs201389706</td><td>3/4
0/4</td><td>2</td></tr>
</table>

SETP9 p.P29T

Interpretation: Variants in this gene is associated with HNA AD form, onset in 1st to 3rd decade, episodes of pain followed by weakness and atrophy, neuropathy and facial dysmorphic features. Variants in this gene are also associated with cancer. The variant is predicted pathogenic in only one VPD, situated in a minor conserved area. Her phenotype included cervicobrachialgia at 51 years of age and 12 years later symptomatology from lower extremities. The genotype does not correlate with the phenotype of this patient.

Classification: Unlikely pathogenic

PRX p.K162T + p.V871A

Interpretation: Variants in this gene are associated with DSN AR type. The p.K162T variant is predicted pathogenic but is present as heterozygous. The p.V871A variant is too common to be pathogenic as compound heterozygous. Nor does the phenotype of this patient and age of onset correlate with DSN.

Classification: Unlikely pathogenic

27 (178)	M	-	CMT	D	*DCTN1*	c.1951C>T	p.R651W	NM_001135041.2	het	-	-/0.07	rs121909344	4/4	3
					LMNA	c.1840C>T	p.R614C	NM_170708.3	het	-	-/0.11	rs142000963	4/4	4

DCTN1 p.R651W

Interpretation: Variants in this gene are associated with dHMN AD form, onset in early adulthood, slow progression, facial weakness vocal cord paralysis and breathing difficulty. This variant has been reported pathogenic as heterozygous in two family members with ALS but the variant was also present in two healthy family members, the authors suggested incomplete penetrance [8]. This variant is also present in ESP at low frequency. Phenotype of reported individuals does not correlate with phenotype in this patient. It can thus be debated whether this variant is a pathogenic variant.

Classification: Uncertain pathogenic

LMNA p.R614C

Interpretation: Variants in LMNA have been associated with a broad range of phenotypes, broadly classified as 1) neuromuscular and cardiac disorders and 2) lipodystrophy and premature aging disorders [9,10]. Several variants associated with CMT2 have been identified, both autosomal dominant and recessive inheritance [9]. The variant identified in this family and also another CMT family (family 54) has been classified as a variant of unknown significance due to extreme phenotypic diversity including reports of motor neuropathy and also low penetrance in affected families [11]. A previous published report argue however that there is strong evidence in support of its pathogenicity, the variant has been found in 19 patients and not in 1000 controls (including two patients in our material and 250 controls), the residue is totally conserved and studies of fibroblast carrying this variant show abnormalities of nuclear shape [11]. The variant was however found in the ESP database at 0,1%, but it has to be taken into account that the ESP database contain selected affected and controls for specific traits as LDL, blood pressure, early onset myocardial infarction, early stroke, and lung disease, traits that have been associated with *LMNA* mutations. The strongest hypothesis for the nonpenetrance and variable expressivity of this variant is the existence of a pathogenic variant in another gene giving dual pathology, which has previously been observed in three cases [11-13]. Intriguingly, both our cases carried another variant classified uncertain pathogenic. It was not possible to obtain the neurophysiology for this patient but the patient in family 54 has CMT2 which match previous reported phenotypes for variants in this gene.

	Classification: Likely pathogenic<br><br>*LAMNA* and *DCTN1* are situated in the same molecular pathway, activation of chaperone genes by *XBP1*, a transcription factor associated with differentiation of neurons [14],[footnote 5]. It is however uncertain whether this might be the possible disease mechanism.													
32 (165)	M	35	CMT2	AD	*PRX*	c.731C>T<br>c.3373G>A	p.A244V<br>p.G1125S	NM_181882.2	comp. het	10<br>3	1.00/1.14<br>-/0.24	rs118071705<br>rs148939995	1/4<br>2/4	1
	Interpretation: Variants in this gene are associated with DSN AR type. Genotype correlates not with the phenotype of patient and inheritance pattern in family, these variants are also too common even as compound heterozygous to be pathogenic.<br><br>Classification: Certainly not pathogenic													
34 (209)	M	60	CMT2	D	No variants of probable clinical relevance									
37 (200)	F	-	CMT2	D	No variants of probable clinical relevance									
37 (217)	M	39	CMT2	D	No variants of probable clinical relevance									
41 (223)	F	15	CMT2	-	*SETX*	c.59G>A<br>c.3809C>T	p.R20H<br>p.P1270L	NM_015046.5	comp. het	1<br>-	0.64/0.73<br>0.05/0.05	rs79740039<br>rs144334281	0/4<br>1/4	3
	Interpretation: Heterozygous variants in *SETX* are associated with ALS, dHMN, and tremor-ataxia syndrome. Homozygous or compound heterozygous variants have been associated with AOA2 and spinocerebellar ataxia. The p.R20H variant is predicted benign, minor conserved and relatively frequent. The p.P127L variant is predicted pathogenic in one VPD, highly conserved and situated in close proximity to another variant associated with ataxia with neuropathy as homozygous. Most other variants in this exon are associated with AOA2. Considering the fact that these two variants also were observed in another patient with CMT in our clinic, the p.R20H variant was found in only one in-house control and the p.P1270L variant was absent from in-house controls, we are uncertain whether the compound heterozygote could be pathogenic.<br><br>Classification: Uncertain pathogenic as compound heterozygous.													
45 (261)	F	9	CMT2	Sporadic	*SH3TC2*	c.1298C>T	p.S433L	NM_024577.3	het	2	-/0.0007	-	3/4	1
	Interpretation: Variants in this gene are associated with CMT1 AR type, variable onset and phenotype. The reported phenotype correlates not with the phenotype of this patient, and this variant is also seen in the patient's unaffected father and in another patient with CMT1 (family 7) and in his likely unaffected child.<br><br>Classification: Certainly not pathogenic													
46 (258)	M	63	CMT1	AD	*PRX*	c.2260C>T	p.R754W	NM_181882.2	het	-	-/0.02	-	2/4	2
	Interpretation: Variants in this gene are associated with DSN and CMT1 AR type. This heterozygous variant is not very frequent, well conserved and predicted pathogenic in two VPD. The variant													

<table>
<tr><td></td><td colspan="14">was however classified unlikely pathogenic as only homozygous and compound heterozygous variants have been reported for this gene, other heterozygous variants in this gene are frequently found among the controls and the patient has later onset than more mildly decreased NCV (33m/s) than most other reported cases.

Classification: Unlikely pathogenic</td></tr>
<tr><td rowspan="3">54 (328)</td><td rowspan="2">M</td><td rowspan="2">56</td><td rowspan="2">CMT2</td><td rowspan="2">Sporadic</td><td>ARHGEF10</td><td>c.2197C>T</td><td>p.H733Y</td><td>NM_014629.2</td><td>het</td><td>-</td><td>0.05/0.13</td><td>rs147531758</td><td>1/4</td><td>3</td></tr>
<tr><td>LMNA</td><td>c.1840C>T</td><td>p.R614C</td><td>NM_170708.3</td><td>het</td><td>-</td><td>-/0.11</td><td>rs142000963</td><td>4/4</td><td>4</td></tr>
<tr><td colspan="14">ARHGEF10 p.H733Y

Interpretation: One variant in ARHGEF10 have been associated with slow nerve conduction in AD form [15]. This variant is predicted pathogenic in one VPD, it is partly conserved and gives a large amino acid change. This variant is also present in three individuals with CMT2 in this material (family 54 and 282) and not seen in any in-house controls, but present in the ESP database at 0.13%.

Classification: Uncertain pathogenic

LMNA p.R614C

Interpretation: Variants in LMNA have been associated with a broad range of phenotypes, broadly classified as 1) neuromuscular and cardiac disorders and 2) lipodystrophy and premature aging disorders [9,10]. Several variants associated with CMT2 have been identified, both autosomal dominant and recessive inheritance [9]. The variant identified in this family and also another CMT family (family 27) has been classified as a variant of unknown significance due to extreme phenotypic diversity including reports of motor neuropathy and also low penetrance in affected families [11]. A previous published report argue however that there is strong evidence in support of its pathogenicity, the variant has been found in 19 patients and not in 1000 controls (including two patients in our material and 250 controls), the residue is totally conserved and studies of fibroblast carrying this variant show abnormalities of nuclear shape [11]. The variant was however found in the ESP database at 0,1%, but it has to be taken into account that the ESP database contain selected affected and controls for specific traits as LDL, blood pressure, early onset myocardial infarction, early stroke, and lung disease, traits that have been associated with LMNA mutations. The strongest hypothesis for the nonpenetrance and variable expressivity of this variant is the existence of a pathogenic variant in another gene giving dual pathology, which has previously been observed in three cases [11-13]. Intriguingly, both our cases carried another variant classified uncertain pathogenic. This patient has CMT2 which match previous reported phenotypes for variants in this gene.

Classification: Likely pathogenic

ARHGEF10 and LMNA are both involved in myelination and cell morphology [15,16], [footnote 5]. Thus a dual pathologic effect of these two variants might be likely.</td></tr>
<tr><td>55 (307)</td><td>F</td><td>6</td><td>CMT</td><td>Sporadic</td><td colspan="10">No variants of probable clinical relevance</td></tr>
<tr><td rowspan="2">62 (318)</td><td>M</td><td>33</td><td>CMT2</td><td>Sporadic</td><td>POLG</td><td>c.1491G>C

c.2243G>C</td><td>p.Q497H

p.W748S</td><td>NM_001126131.1</td><td>comp. het</td><td>-</td><td>-/-</td><td>rs121918052

rs113994097</td><td>3/4

2/4</td><td>4</td></tr>
<tr><td colspan="14">Interpretation: These two variants have been reported to cause ataxia and additional features of headache, epilepsy, myoclonus, neuropathy and late onset PEO in two Norwegian patients as compound homozygous. The two variants were however present in a compound heterozygous state in a family member of one of the patients, in one control, and in one other patient [17]. The variant W748S have also been reported to cause neurodegenerative disorders with ataxia in three patients and dHMN in five patients as compound heterozygous or compound homozygous [18,19]. The controls and family members in the Norwegian study was not investigated for purely neuropathy but for a more serious phenotype making it likely that a neuropathy phenotype could have been overlooked Mutations in POLG have also been reported to cause axonal CMT [20] which correlates well with the phenotype of this patient. As this patient is a sporadic case, compound</td></tr>
</table>

	heterozygous mutations are a likely cause. These variants might cause a lighter phenotype with neuropathy when present as compound heterozygous. These variants was neither seen in our in-house controls nor present in SNP databases. These two variants are also present in another patient in our clinic with similar phenotype.<br><br>Classification: Likely pathogenic													
65 (296)	M	36	CMT2	Sporadic	No variants of probable clinical relevance									
78 (426)	M	20	CMT	D	*GAN*	c.730A>G	p.I244V	NM_022041.3	het	-	-/0.04	rs200749953	1/4	3
					PRX	c.823C>A	p.L275I	NM_181882.2	het	-	-/0.07	rs200033507	2/4	1
	GAN p.I244V<br><br>Interpretation: Mutations in *GAN* causes peripheral motor and sensory neuropathy, foot deformities and giant axonal swelling, CMT2 phenotypes have also been reported [21,22]. Variants occur mostly in AR form, but AD forms and heterozygous carriers with progressive neurological condition and light neuropathy have also been reported [23-26]. This heterozygous variant is predicted benign in three VPD and creates a minor amino acid change, it is relatively well conserved and placed in close proximity to another pathogenic variant that is present as homozygous and creates a stop in the protein [25]. The variant is however not present in the patient's unaffected brother and niece, not seen in any controls, present in the ESP at low frequency (0,04%).<br><br>Classification: Uncertain pathogenic<br><br>*PRX* p.L275I<br><br>Interpretation: Variants in this gene are associated with CMT1 and DSN AR type. This patient has a heterozygous variant, the variant is present in the patient's unaffected brother and niece, and also in family 6 where it neither segregated with the phenotype.<br><br>Classification: Certainly not pathogenic													
84 (468)	F	48	CMT2	D	No variants of probable clinical relevance									
90 (507)	F	2	CMT1	AR	*MFN2*	c.310C>T	p.R104W	NM_001127660.1	het	-	-/-	rs119103268	4/4	5
	Interpretation: This variant has been reported to cause early onset severe CMT2 in several reports and have been described as a mutational hotspot [28-30]. This patient was severely affected and had slightly decreased motor NCV, 36 m/s, genotype correlates with phenotype. Her parents were related, third or fourth cousins, thus AR inheritance was first assumed. It is uncertain why this mutation was not discovered in the previous study by Braathen et al [3].<br><br>Classification: Certainly pathogenic													
95 (534)	M	12	CMT2	Sporadic	*IGHMBP2*	c.165G>C	p.Q55H	NM_002180.2	het	-	-/0.04	rs201692151	3/4	1
					REEP1	c.524A>G	p.*175Trpext*55	NM_001164731.1	het	-	-/-	-	n.a.	4

<table>
<tr><td rowspan="2"></td><td></td><td></td><td></td><td></td><td>SETX</td><td>c.3075_3076insTGA</td><td>p.R1026*</td><td>NM_015046.5</td><td>het</td><td>-</td><td>-/-</td><td>-</td><td>n.a.</td><td>4</td></tr>
<tr><td colspan="14">IGHMBP2 p.Q55H

Interpretation: Variants in IGHMBP2 are associated with dHMN AR form, highly severe phenotype and death within 12 months. This is a heterozygous variant, clinical characteristics of this patient is CMT2 and spasticity, genotype does not correlate with phenotype.

Classification: Certainly not pathogenic

REEP1 p.*175Trpext*55

Interpretation: Variants in REEP1 are associated with mainly spastic paraplegia AD form but also dHMN AD form. This novel variant is heterozygous and causes a stop loss mutation extending the protein by 55 amino acids. As this patient also showed spastic signs, we consider this variant to cause the spasticity not the CMT phenotype.

Classification: Likely pathogenic

SETX p.R1026*

Heterozygous variants in SETX are associated with ALS, dHMN, and tremor-ataxia syndrome. Homozygous or compound heterozygous variants have been associated with AOA2 and spinocerebellar ataxia. This variant creates a premature stop codon at position 1026 of totally 2678 amino acids. The genotype correlates well with phenotype as dHMN often overlaps with CMT2

Classification: Likely pathogenic

Thus, we assume digenic pathogenicity; the SETX variant causing CMT2 and the REEP1 variant causing spasticity.</td></tr>
<tr><td rowspan="2">102 (561)</td><td>M</td><td>50</td><td>CMT2</td><td>AD</td><td>HSPB1</td><td>c.380G>T</td><td>p.R127L</td><td>NM_001540.3</td><td>het</td><td>-</td><td>-/-</td><td>-</td><td>3/4</td><td>5</td></tr>
<tr><td colspan="14">Interpretation: Variants in this gene are associated with both CMT2 and dHMN AD forms, variable onset, slowly progressive, fasciculations and muscle cramps. A variant in the same triplet as our variant has been reported pathogenic as a heterozygous Arginine to Tryptophan substitution in several patients with CMT2 and dHMN and also confirmed in cellular studies [31-34]. This variant is a heterozygous Arginine to Leucine substitution at the same position, creating a large change in amino acid structure and predicted pathogenic. The genotype segregates with the family phenotype, present in two affected members (patient 561 and 689) and not in a third unaffected family member.

Classification: Certainly pathogenic</td></tr>
<tr><td rowspan="2">102 (689)</td><td>F</td><td>36</td><td>CMT2</td><td>AD</td><td>HSPB1</td><td>c.380G>T</td><td>p.R127L</td><td>NM_001540.3</td><td>het</td><td>-</td><td>-/-</td><td>-</td><td>3/4</td><td>5</td></tr>
<tr><td colspan="14">Interpretation: See patient 561 for interpretation of this variant

Classification: Certainly pathogenic</td></tr>
<tr><td>104 (583)</td><td>M</td><td>65</td><td>CMT2</td><td>AR</td><td colspan="10">No variants of probable clinical relevance</td></tr>
</table>

<table>
<tr><td>105 (586)</td><td>M</td><td>45</td><td>CMT1</td><td>D</td><td colspan="10">No variants of probable clinical relevance</td></tr>
<tr><td>111 (589)</td><td>M</td><td>39</td><td>CMT2</td><td>Sporadic</td><td colspan="10">No variants of probable clinical relevance</td></tr>
<tr><td rowspan="2">112 (597)</td><td>M</td><td>48</td><td>CMT2</td><td>AR</td><td>GAN</td><td>c.23C>G</td><td>p.S8C</td><td>NM_022041.3</td><td>het</td><td>-</td><td>-/-</td><td>-</td><td>1/4</td><td>3</td></tr>
<tr><td colspan="14">Interpretation: Mutations in GAN causes peripheral motor and sensory neuropathy, foot deformities and giant axonal swelling, CMT2 phenotypes have also been reported [21,22]. Variants occur mostly in AR form but AD forms and heterozygous carriers with progressive neurological condition and light axonal neuropathy have also been reported [23-26]. This novel heterozygous variant is predicted benign in three VPD, but the variant creates a large change in amino acid physiochemical properties, it is highly conserved, situated in the Kelch-like protein, gigaxonin domain, and placed in close proximity to other pathogenic variants associated with GAN as homozygous or compound heterozygous [23,24,35]. The inheritance pattern was assumed AR as there are two affected brothers in this family with unaffected parents, thus there might be an additional variant that has not been identified. DNA was not obtained from the brother.

Classification: Uncertain pathogenic</td></tr>
<tr><td>114 (600)</td><td>M</td><td>30</td><td>CMT2</td><td>AD</td><td colspan="10">No variants of probable clinical relevance</td></tr>
<tr><td>114 (668)</td><td>M</td><td>16</td><td>CMT2</td><td>AD</td><td colspan="10">No variants of probable clinical relevance</td></tr>
<tr><td rowspan="3">123 (616)</td><td rowspan="2">M</td><td rowspan="2">24</td><td rowspan="2">CMT2</td><td rowspan="2">D</td><td>KIF1B</td><td>c.881A>G</td><td>p.K294R</td><td>NM_015074.3</td><td>het</td><td>-</td><td>-/0.01</td><td>-</td><td>3/4</td><td>4</td></tr>
<tr><td>SBF2</td><td>c.5020_5022del</td><td>p.Glu1674del</td><td>NM_030962.3</td><td>het</td><td>1</td><td>-/-</td><td>-</td><td>n.a.</td><td>1</td></tr>
<tr><td colspan="14">KIF1B p.K294R

Interpretation: This variant was totally conserved, predicted pathogenic, situated in the kinesin motor domain, and found among one individual in the ESP database (0,008%). This variant was situated in the same highly conserved domain as a heterozygous variant (Gln98Leu) reported in another CMT2 family. [36]. Researchers have been cautious about classifying KIF1B, which is a reterograde transport protein, a CMT causing gene since only one family has been reported. As functional studies of the previously reported variant have confirmed loss of motor activity and variants in other motorproteins (KIF1A, DYNC1H1, and DCTN1) also are involved in neuropathy [9], we consider KIF1B a possible CMT causing gene and classify our variant likely pathogenic. DNA was only available from one case in this family.

Classification: Likely pathogenic

SBF2 p.Glu1674del

Interpretation: Variants in this gene are associated with CMT1 AR type severe form. This variant is a heterozygous deletion of Glutamic acid, an unconserved amino acid at this position. Reported phenotype does not correlate with phenotype of this patient.

Classification: Certainly not pathogenic.</td></tr>
<tr><td>126 (619)</td><td>F</td><td>12</td><td>CMT1</td><td>AD</td><td colspan="10">No variants of probable clinical relevance</td></tr>
</table>

142 (638)	F	1	CMT1	Sporadic	*POLG*	c.2740A>C	p.T914P	NM_001126131.1	het	-	-/0.02	rs139590686	4/4	1
					SH3TC2	c.2860C>T	p.R954X	NM_024577.3	hom	5	-/0.08	rs80338933	n.a.	5
	POLG p.T914P<br><br>Interpretation: This variant has been reported to cause late onset PEO, encephalopathy, and myopathy in a patient as compound heterozygous (p.Q308H + p.T914P), the variant p.T914P was not seen in any of 864 controls [37]. Another group reported this variant to cause MSCAE as compound heterozygous in a patient (p.T914P + p.W748S) [38]. This patient presents with a severe CMT1 phenotype, age of onset was 6 months and the variant is present in a heterozygous state, genotype does not correlate with phenotype. This variant is also present in the patient's unaffected mother.<br><br>Classification: Certainly not pathogenic<br><br>*SH3TC2* p.R954X<br><br>Interpretation: This variant is well documented to cause CMT1 AR form as homozygous or compound heterozygous, and results in a premature stop in the protein at position 954 of totally 1289 amino acids [39-42]. The reported phenotype is highly variable even within the same family with onset in 1st to 4th decade variable severity and often scoliosis. This girl has been operated for scoliosis and the R954X variant presented as homozygous, thus genotype correlates well with phenotype of this patient. This variant was also present as heterozygous in three unaffected family members, including her unrelated parents, and in five in-house controls.<br><br>Classification: Certainly pathogenic													
146 (919)	F	20	CMT1	D	No variants of probable clinical relevance									
154 (661)	F	5	CMT1	Sporadic	*HSPB3*	c.271G>A	p.E91K	NM_006308.2	het	-	-/0.03	rs147724326	4/4	1
	Interpretation: Variants in *HSPB3* have been associated with dHMN AD form. This heterozygous variant was predicted pathogenic in all VPD, gave a large amino acid change and the amino acid was highly conserved. The patient presented however with a CMT1 phenotype and the variant was also seen in three unaffected family members.<br><br>Classification: Certainly not pathogenic													
159 (675)	M	59	CMT1	AD	*SETX*	c.1015A>C	p.K339Q	NM_015046.5	het	-	-/-	-	1/4	2
	Interpretation: Heterozygous variants in *SETX* are associated with ALS, dHMN, and tremor-ataxia syndrome. Homozygous or compound heterozygous variants have been associated with AOA2 and spinocerebellar ataxia. This heterozygous variant is predicted benign in three VPD and is minor conserved, the phenotype of this patient, CMT1, does not correlate with the reported phenotype dHMN/light ALS. This variant is situated in close proximity to a variant associated with AOA2.<br><br>Classification: Unlikely pathogenic													
162 (680)	M	5	CMT1	AD	*SH3TC2*	c.3550A>G	p.M1184V	NM_024577.3	het	-	-/0.02	rs142451273	3/4	3
	Interpretation: Variants in *SH3TC2* are associated with CMT1 AR form, onset in 1st to 4th decade with highly variable phenotype. This variant was predicted pathogenic in three VPD, totally													

	conserved, situated in the protein domains Tetratricopeptide TPR-1 and tetratricopeptide repeat and in close proximity to other pathogenic variants. The phenotype of the patient correlates with the genotype apart from the presence of a heterozygous variant. However it has been speculated whether some heterozygous variants might be pathogenic[4] and heterozygous carriers have been observed with patchy axonal neuropathy (p.Y169H) and mild mononeuropathy (p.R954X) [43]. In this study the *SH3TC2* p.R954X variant was observed as heterozygote among ten family members, of which two (patient 891 and 941) presented with mild neuropathy. Also another CMT1 family in this material (family 215) presented with a novel heterozygous variant in this gene, predicted pathogenic and highly conserved. Thus, it remains uncertain whether this variant could be pathogenic.<br><br>Classification: Uncertain pathogenic													
169 (696)	F	60	CMT2	Sporadic	No variants of probable clinical relevance									
173 (703)	F	61	CMT2	AR	No variants of probable clinical relevance									
	Parents were third cousins													
174 (704)	M	7	CMT1	D	No variants of probable clinical relevance									
185 (740)	M	15	CMT2	Sporadic	No variants of probable clinical relevance									
186 (746)	M	61	CMT1	Sporadic	*SBF2*	c.5020_5022del	p.E1674del	NM_030962.3	het	1	-/-	-	n.a.	2
	Interpretation: Variants in *SBF2* are associated with CMT1 AR for and are characterized by onset in 1st or 2nd decade, severe distal sensory impairment and glaucoma. This variant, a heterozygous deletion of Glutamic acid is situated in a minor conserved area and is also present in one control. This patient has a CMT1 phenotype but the variant is presented as heterozygous and the age at onset is in sixth decade.<br><br>Classification: Unlikely pathogenic													
198 (758)	M	10	CMT2	D	*IGHMBP2*	c.2360C>T	p.P787L	NM_002180.2	het	-	0.09/0.03	rs141594765	0/4	1
	Interpretation: Variants in this gene are associated with dHMN AR type, onset within first 3 months, death within 12 months, severe phenotype. This variant is heterozygous, predicted benign and present at 0.09% in 1000 genomes, no genotype-phenotype correlation.<br><br>Classification: Certainly not pathogenic													
204 (760)	F	62	CMT1	D	*SEPT9*	c.517G>A	p.A173T	NM_001113493.1	het	-	-/0.01	rs199861986	0/4	1
	Interpretation: Variants in this gene are associated with HNA AD type, onset in 1st to 3rd decade, episodes of pain followed by weakness and atrophy, neuropathy and facial dysmorphic features. The variant is predicted benign in 4 VPD and is minor conserved. No genotype-phenotype correlation.<br><br>Classification: Certainly not pathogenic													
205 (761)	F	-	CMT	Sporadic	*GDAP1*	c.106+6del	-	NM_001040875.2	het	-	-/-	-	n.a	2

<table>
<tr><td></td><td colspan="14">Interpretation: Variants in this gene have been associated with axonal CMT both AD and AR form, the AR type being more severe and lower age at onset. This variant is a deletion of glycine six bp after the end of exon 2. This variant is only predicted pathogenic by one splice site predictor (Human Splicing Finder) and is minor conserved. [footnote 4]

Classification: Unlikely pathogenic</td></tr>
<tr><td rowspan="3">215 (784)</td><td rowspan="2">F</td><td rowspan="2">40</td><td rowspan="2">CMT1</td><td rowspan="2">D</td><td>SH3TC2</td><td>c.1342G>C</td><td>p.D448H</td><td>NM_024577.3</td><td>het</td><td>1</td><td>-/-</td><td>-</td><td>3/4</td><td>3</td></tr>
<tr><td>TRPV4</td><td>c.431C>G</td><td>p.P144R</td><td>NM_001177428.1</td><td>het</td><td>-</td><td>-/-</td><td>-</td><td>1/4</td><td>2</td></tr>
<tr><td colspan="14">SH3TC2 p.D448H

Interpretation: Variants in SH3TC2 are associated with CMT1 AR form, onset in 1st to 4th decade with highly variable phenotype. This novel variant was predicted pathogenic in three VPD, highly conserved, not observed in any SNP databases or in-house controls and situated in the same exon as other pathogenic variants. The phenotype of the patient correlates with the genotype apart from the presence of a heterozygous variant. However it has been speculated whether some heterozygous variants might be pathogenic [4] and heterozygous carriers have been observed with patchy axonal neuropathy (p.Y169H) and mild mononeuropathy (p.R954X) [43]. In this study the p.R954X variant was observed as heterozygote among ten family members, of which two (patient 891 and 941) presented with mild neuropathy. Also another CMT1 family in this material (family 162) presented with a heterozygous variant in this gene, predicted pathogenic and totally conserved. Thus, it remains uncertain whether this variant could be pathogenic.

Classification: Uncertain pathogenic

TRPV4 p.P144R

Interpretation: Variants in TRPV4 are associated with CMT2 AD form, variable onset, phenotypic variability, vocal cord, respiratory and bladder involvement, hearing loss and scoliosis. Variants in this gene are also associated with other skeletal disorders. This variant is predicted pathogenic in one VPD but it is well conserved and gives a large amino acid change. However there is no genotype-phenotype correlation.

Classification: Unlikely pathogenic

SH3TC2 and TRPV4 are not connected in any molecular processes or pathways. [footnote 5]</td></tr>
<tr><td>218 (793)</td><td>M</td><td>52</td><td>CMT1</td><td>Sporadic</td><td colspan="10">No variants of probable clinical relevance</td></tr>
<tr><td rowspan="3">231 (814)</td><td rowspan="2">M</td><td rowspan="2">54</td><td rowspan="2">CMT2</td><td rowspan="2">AD</td><td>DYNC1H1</td><td>c.1700G>A</td><td>p.R567H</td><td>NM_001376.4</td><td>het</td><td>-</td><td>-/-</td><td>-</td><td>1/4</td><td>4</td></tr>
<tr><td>GAN</td><td>c.1084G>A</td><td>p.E362K</td><td>NM_022041.3</td><td>het</td><td>-</td><td>-/-</td><td>-</td><td>1/4</td><td>3</td></tr>
<tr><td colspan="14">DYNC1H1 p.R567H

Interpretation: One family with CMT2 has so far been identified with a pathogenic heterozygous variant in this gene. The phenotype in the reported family was CMT2 AD form, onset in childhood, slowly progressive and variable severity [44]. A few other variants in this gene have been associated with SMA and mental retardation. This novel variant is predicted pathogenic in one VPD, but</td></tr>
</table>

<table>
<tr><td></td><td colspan="14">the variant is highly conserved and is situated in the same domain as the other variant reported to cause CMT2. Apart from higher age of onset in this patient, the phenotype and inheritance correlates well with the family previously reported.

Classification: Likely pathogenic

GAN p.E362K

Interpretation: Mutations in *GAN* causes peripheral motor and sensory neuropathy, foot deformities and giant axonal swelling, CMT2 phenotypes have also been reported [21,22]. Variants occur mostly in AR form but AD forms and heterozygous carriers with progressive neurological condition and light neuropathy have also been reported [23-26]. This novel heterozygous variant is predicted benign in three VPD, but the variant creates a large change in amino acid physiochemical properties, it is highly conserved, situated in the domains Kelch repeat type 1 and Kelch-like protein, gigaxonin, and placed in close proximity to other pathogenic variants associated with GAN as compound heterozygous [27,35].

Classification: Uncertain pathogenic

DYNC1H1 and *GAN* are not connected in any molecular processes or pathways. [footnote 5]</td></tr>
<tr><td rowspan="2">232 (815)</td><td>M</td><td>1</td><td>CMT2</td><td>AD</td><td>*MTMR2*</td><td>c.810A>C</td><td>p.L270F</td><td>NM_016156.5</td><td>het</td><td>-</td><td>-/-</td><td>-</td><td>4/4</td><td>3</td></tr>
<tr><td colspan="14">Interpretation: Variants in this gene are associated with CMT1 AR form, onset 1st decade, death in 4th to 5th decade and severe course. There are a few examples of dominant pathogenic variants in this gene as well [45,46]. This heterozygous variant is predicted pathogenic in all VPD, totally conserved, situated in the myotubularin domain in close proximity to three other variants associated with CMT which causes disease as heterozygous, homozygous, or compound heterozygous [45-47], not seen in any controls. It is also noteworthy that among the 180 in-house controls only one heterozygous variant was observed in this gene (p.I281V), predicted benign and minor conserved. Whereas three patients (family 232 and 404) had heterozygous variants in this, gene predicted pathogenic and well conserved. It could thus be debated whether variants in MTMR2 could cause disease as heterozygous resulting in a lighter phenotype than observed with homozygous variants. This patient had however CMT2, not CMT1 as usually reported for variants in this gene.

Classification: Uncertain pathogenic</td></tr>
<tr><td rowspan="4">252 (843)</td><td rowspan="3">F</td><td rowspan="3">3</td><td rowspan="3">CMT1</td><td rowspan="3">D</td><td>*AARS*</td><td>c.497T>G</td><td>p.I166S</td><td>NM_001605.2</td><td>het</td><td>-</td><td>-/0.02</td><td>rs199997425</td><td>4/4</td><td>3</td></tr>
<tr><td>*ARHGEF10*</td><td>c.2063G>A</td><td>p.S688N</td><td>NM_014629.2</td><td>het</td><td>-</td><td>-/0.07</td><td>rs143290224</td><td>0/4</td><td>2</td></tr>
<tr><td>*SH3TC2*</td><td>c.2860C>T</td><td>p.R954X</td><td>NM_024577.3</td><td>hom</td><td>5</td><td>-/0.08</td><td>rs80338933</td><td>n.a.</td><td>5</td></tr>
<tr><td colspan="14">*AARS* p.I166S

Interpretation: Variants in *AARS* are associated with CMT2 AD form, variable onset and variable severity. This variant is predicted pathogenic in all VPD, and situated in same domain as other pathogenic variants. Variants in this gene are however associated with an axonal CMT and this patient presents with a demyelinating CMT. The patient also has another variant that is certain pathogenic that matches the phenotype. It is uncertain whether this variant could contribute to the phenotype.

Classification: Uncertain pathogenic

ARHGEF10 p.S688N</td></tr>
</table>

<table>
<tr><td></td><td colspan="14">Interpretation: One variant in this gene has been associated with slow nerve conduction AD form [15]. This heterozygous variant is predicted benign, situated in a minor conserved area, and in close proximity to other benign variants. This variant is also reported in ESP at 0.07%.

Classification: Unlikely pathogenic

SH3TC2 p.R954X

Interpretation: This variant is well documented to cause CMT1 AR form as homozygous or compound heterozygous, and results in a premature stop in the protein at position 954 of totally 1289 amino acids [39-42]. The reported phenotype is highly variable even within the same family with onset in 1st to 4th decade variable severity and often scoliosis. This girl has light scoliosis and the R954X variant presented as homozygous, thus genotype correlates well with phenotype of this patient. Dominant inheritance was first assumed for the family as the mother had a mild neuropathy.

Classification: Certainly pathogenic</td></tr>
<tr><td rowspan="3">252 (891)</td><td rowspan="2">F</td><td rowspan="2">5</td><td rowspan="2">Neuro-pathy</td><td rowspan="2">D</td><td>*ARHGEF10*</td><td>c.2063G>A</td><td>p.S688N</td><td>NM_014629.2</td><td>het</td><td>-</td><td>-/0.07</td><td>rs143290224</td><td>0/4</td><td>2</td></tr>
<tr><td>*SH3TC2*</td><td>c.2860C>T</td><td>p.R954X</td><td>NM_024577.3</td><td>het</td><td>5</td><td>-/0.08</td><td>rs80338933</td><td>n.a.</td><td>3</td></tr>
<tr><td colspan="14">*ARHGEF10* p.S688N

Interpretation: One variant in this gene has been associated with slow nerve conduction AD form. This heterozygous variant is predicted benign, is situated in a minor conserved area, and in close proximity to other benign variants. This variant is also reported in ESP at 0.07%.

Classification: Unlikely pathogenic

SH3TC2 p.R954X

Interpretation: This variant is well documented to cause CMT1 AR form as homozygous or compound heterozygous, and results in a premature stop in the protein at position 954 of totally 1289 amino acids [39-42]. The reported phenotype is highly variable even within the same family with onset in 1st to 4th decade variable severity and often scoliosis. Although this gene is reported as a AR gene, it has been speculated whether some heterozygous variants might be pathogenic [4], and heterozygous carriers have been observed with patchy axonal neuropathy (p.Y169H) and mild mononeuropathy (p.R954X) [43]. The p.R954X was present in our material among five in-house controls and 10 family members of which two presented with mild neuropathy. This patient had recurrent ankle sprains and unsteadiness from 5 years of age and at 70 years of age she had symptomatology consistent with a mild neuropathy. It is uncertain whether this variant could contribute to her phenotype.

Classification: Uncertain pathogenic</td></tr>
<tr><td rowspan="3">257 (860)</td><td rowspan="2">F</td><td rowspan="2">38</td><td rowspan="2">CMT2</td><td rowspan="2">Sporadic</td><td>*ARHGEF10*</td><td>c.1013G>C</td><td>p.R338T</td><td>NM_014629.2</td><td>het</td><td>-</td><td>-/-</td><td>-</td><td>0/4</td><td>4</td></tr>
<tr><td>*SETX*</td><td>c.7640T>C</td><td>p.I2547T</td><td>NM_015046.5</td><td>het</td><td>-</td><td>-/0.54</td><td>rs1511117904</td><td>0/4</td><td>2</td></tr>
<tr><td colspan="14">*ARHGEF10* p.R338T

Interpretation: This novel variant is highly conserved, and creates an extensive change in amino acid physiochemical properties, but is predicted benign. This variant is also situated in close proximity to another heterozygous variant (Thr332Ile) associated with decreased NCV and thin myelination.[15] Functional studies show that the Thr332Ile mutant stimulates increased actomyosin</td></tr>
</table>

	contraction, regulating cell morphology in Schwann cells [48]. This sporadic case had NCV in the same range as the previous reported family.<br><br>Conclusion: Likely pathogenic<br><br>*SETX* p.I2547T<br><br>Interpretation: Heterozygous variants in *SETX* are associated with ALS, dHMN, and tremor-ataxia syndrome. Homozygous or compound heterozygous variants have been associated with AOA2 and spinocerebellar ataxia. This variant has been reported pathogenic as heterozygous in one patient with tremor ataxia and mild motor neuron signs [6]. This variant is however also relatively common and present in ESP at 0.5% and it is also present in another patient with a CMT1 phenotype (family 10).<br><br>Classification: Unlikely pathogenic													
277 (880)	F	46	CMT1	D	No variants of probable clinical relevance									
282 (886)	F	5	CMT2	D	*ARHGEF10*	c.2197C>T	p.H733Y	NM_014629.2	het	-	-/0.13	rs147531758	1/4	3
					SLC12A6	c.1610G>A	p.G537D	NM_001042497.1	het	-	-/-	-	1/4	1
	ARHGEF10 p.H733Y<br><br>Interpretation: One variant in *ARHGEF10* have been associated with slow nerve conduction in AD form [15]. This variant is predicted pathogenic in one VPD, it is partly conserved and creates a large amino acid change. We consider it uncertain whether this variant could contribute to the phenotype as this variant was present in three affected patients in this material (family 54 and 282) and not seen in any in-house controls. This variant was however present in the ESP database at 0.13%.<br><br>Classification: Uncertain pathogenic<br><br>*SLC12A6* p.G537D<br><br>Interpretation: Variants in this gene are associated with ACCPN AR form, onset in infancy, progressive, death in 3rd to 4th decade, peripheral motor and sensory neuropathy, demyelinating and axonal, delayed motor milestones, facial dysmorphic features, scoliosis, developmental delay, seizures. This variant is present as heterozygous and genotype does not correlate with phenotype. The variant is not present in the sister, patient 903.<br><br>Classification: Certainly not pathogenic													
282 (903)	F	6	CMT2	D	*ARHGEF10*	c.2197C>T	p.H733Y	NM_014629.2	het	-	0.05/0.13	rs147531758	1/4	3
	Interpretation: See discussion for patient 886<br><br>Classification: Uncertain pathogenic													
285 (889)	F	5	CMT1	D	*SH3TC2*	c.2860C>T	p.R954X	NM_024577.3	hom	5	-/0.0008	rs80338933	n.a.	5
	Interpretation: This variant is well documented to cause CMT1 AR form as homozygous or compound heterozygous, and results in a premature stop in the protein at position 954 of totally 1289													

	amino acids [39-42]. The reported phenotype is highly variable even within the same family with onset in 1st to 4th decade variable severity and often scoliosis. Scoliosis from 11 years of age. The R954X variant presented as homozygous, thus genotype correlates well with phenotype of this patient. Dominant inheritance was first assumed for the family as the mother had a mild neuropathy.<br><br>Classification: Certainly pathogenic													
285 (941)	F	74	Neuro-pathy	D	*SH3TC2*	c.2860C>T	p.R954X	NM_024577.3	het	5	-/0.08	rs80338933	n.a.	3
	Interpretation: This variant is well documented to cause CMT1 AR form as homozygous or compound heterozygous, and results in a premature stop in the protein at position 954 of totally 1289 amino acids [39-42]. The reported phenotype is highly variable even within the same family with onset in 1st to 4th decade variable severity and often scoliosis. Although this gene is reported as a AR gene, it has been speculated whether some heterozygous variants might be pathogenic [4], and heterozygous carriers have been observed with patchy axonal neuropathy (p.Y169H) and mild mononeuropathy (p.R954X) [43]. The p.R954X was present in our material among five in-house controls and 10 family members of which two presented with mild neuropathy. This patient was unsteady with reduced joint sensibility in first toes at 74 years of age, had slightly reduced nerve conduction velocities and electromyographic findings consistent with a mild neuropathy. It is uncertain whether this variant could contribute to her phenotype.<br><br>Classification: Uncertain pathogenic													
295 (931)	F	2	CMT1	Sporadic	*SH3TC2*	c.2860C>T	p.R954X	NM_024577.3	hom	5	-/0.08	rs80338933	n.a.	5
	Interpretation: This variant is well documented to cause CMT1 AR form as homozygous or compound heterozygous, and results in a premature stop in the protein at position 954 of totally 1289 amino acids [39-42]. The reported phenotype is highly variable even within the same family with onset in 1st to 4th decade variable severity and often scoliosis. This girl had scoliosis, the R954X variant presented as homozygous, thus genotype correlates well with phenotype of this patient.<br><br>Classification: Certainly pathogenic													
320 (946)	M	68	CMT2	D/AR	No variants of probable clinical relevance									
	Four affected sibs and parents not related													
320 (947)	F	40	CMT2	D/AR	No variants of probable clinical relevance									
370 (984)	M	51	CMT2	Sporadic	No variants of probable clinical relevance									
375 (1043)	F	18	CMT1	Sporadic	*SBF2*	c.3831C>G	p.I1277M	NM_030962.3	het	1	-/0.03	rs139522696	1/4	2
	Interpretation: Variants in *SBF2* are associated with CMT1 AR form and are characterized by onset in 1st or 2nd decade, severe distal sensory impairment and glaucoma. This heterozygous variant is minor conserved and is also present in one control.<br><br>Classification: Unlikely pathogenic													
404	F	74	CMT	AD	*MTMR2*	c.*53G>A		NM_016156.5	het	-	-/-	-	n.a.	3

(1030)	Interpretation: Variants in this gene are associated with CMT1 AR form, onset 1st decade, death in 4th to 5th decade and severe course. There are a few examples of dominant pathogenic variants in this gene as well [45,46]. This novel 3'UTR c.*53G>A variant was present in two siblings. The variant was predicted by splice site predictors to change the splice site, the nucleotide was totally conserved. [footnote 4] It is also noteworthy that among the 180 in-house controls only one heterozygous variant was observed in this gene (p.I281V), predicted benign and minor conserved. Whereas three patients (family 232 and 404) had heterozygous variants in this, gene predicted pathogenic and well conserved. It could thus be debated whether variants in MTMR2 could cause disease as heterozygous resulting in a lighter phenotype than observed with homozygous variants. EMG results were not obtainable for this family.<br><br>Classification: Uncertain pathogenic													
404 (1031)	M	47	CMT	AD	*MTMR2*	c.*53G>A		NM_016156.5	het	-	-	-	n.a.	3
	Interpretation: See discussion for patient 1030.<br><br>Classification: Uncertain pathogenic													
412 (1035)	F	33	CMT2	D	No variants of probable clinical relevance									
466 (1022)	F	35	ICMT	AD	*GARS*	c.95T>C	p.L32P	NM_002047.2	het	-	-/-	-	0/4	1
	GARS p.L32P<br><br>Interpretation: Variants in *GARS* are associated with CMT2 and dHMN AD form, most with onset in 2nd decade, slowly progressive and upper-limb predominance This heterozygous variant is predicted benign in four VPD, it is situated in a completely unconserved region with no other pathogenic variants nearby. The variant did not segregate with the phenotype in this family.<br><br>Classification: Certainly not pathogenic													

[1]The family and patient identifiers are not continuous numbered as this material of CMT families, collected from the general population in Eastern Akershus, Norway, is part of a larger material.

[2]The Exome Sequencing Project (ESP) contains data from selected affected and controls for specific traits as LDL, blood pressure, early onset myocardial infarction, early stroke, and lung disease. As some of these individuals probably have neuropathy, our classification system was not based on these frequencies, but rather incorporated these in the interpretation. At the time of interpretation this database contained 6503 individuals.

[3]Variant prediction was performed by SIFT [49], Polyphen, [50], AlignGVGD [51], and Mutation Taster [52] through the Alamut interface v2.2-0 (Interactive Biosoftware, Rouen, France) Prediction only obtainable for non-synonymous exonic variants.

5 Splice site prediction was performed by SpliceSiteFinder [53], MaxEntScan [54], NNSPLICE [55], GeneSplicer [56], and Human Splicing Finder [57] through the Alamut interface v2.2-0.

[5]String-DB [58], Ingenuity Pathway Analysis [59], and Reactome [60] was used to analyze whether genes were connected in the same molecular processes or pathways.

Abbreviations: 1000g = the 1000 Genomes Project; ACCPN=agenesis of the corpus callosum with peripheral neuropathy; AD=autosomal dominant; ALS=amyothropic lateral sclerosis; AOA2=ataxia with occulomotor apraxia; AR=autosomal recessive inheritance; comp; het=compound heterozygous; CMT1=Charcot-Marite-Tooth, demyelinating form; CMT2=Charcot-Marie-Tooth, axonal form;

ICMT=intermediate Charcot-Marie-Tooth; dbSNP= the Single Nucleotide Polymorphism database; D=dominant inheritance; dHMN=distal hereditary motor neuronopathy; DSMAX= X-linked recessive, axonal neuropathy without sensory involvement; DSN= Dejerine-Sottas neuropathy; EMG=electromyography; ESP=the Exome Sequencing Project; F=female; hemi=hemizygous; het=heterozygous; HNA=hereditary neuralgic amyotrophy; hom=homozygous; HSAN= hereditary sensory and autonomic neuropathies; M=male; MSCAE=mitochondrial spinocerebellar ataxia and epilepsy; n.a;=not applicable; NCV=nerve conduction velocity; PEO=progressive external ophthalmoplegia; R=recessive; SMA=spinal muscular athophy; VPD=variant prediction databases; XL=X-linked inheritance; -=no information;

References:

[1] Bone LJ, Deschenes SM, Balice-Gordon RJ, Fischbeck KH, Scherer SS: Connexin32 and X-linked Charcot-Marie-Tooth disease. *Neurobiol Dis* 1997; **4:** 221-230.

[2] Yum SW, Kleopa KA, Shumas S, Scherer SS: Diverse trafficking abnormalities of connexin32 mutants causing CMTX. *Neurobiol Dis* 2002; **11:** 43-52.

[3] Braathen GJ, Sand JC, Lobato A, Hoyer H, Russell MB: Genetic epidemiology of Charcot-Marie-Tooth in the general population. *Eur J Neurol* 2011; **18:** 39-48.

[4] Lassuthova P, Mazanec R, Vondracek P *et al*: High frequency of SH3TC2 mutations in Czech HMSN I patients. *Clin Genet* 2011.

[5] Gallardo E, Claeys KG, Nelis E *et al*: Magnetic resonance imaging findings of leg musculature in Charcot-Marie-Tooth disease type 2 due to dynamin 2 mutation. *J Neurol* 2008; **255:** 986-992.

[6] Hirano M, Quinzii CM, Mitsumoto H *et al*: Senataxin mutations and amyotrophic lateral sclerosis. *Amyotroph Lateral Scler* 2011; **12:** 223-227.

[7] Anheim M, Monga B, Fleury M *et al*: Ataxia with oculomotor apraxia type 2: clinical, biological and genotype/phenotype correlation study of a cohort of 90 patients. *Brain* 2009; **132:** 2688-2698.

[8] Munch C, Sedlmeier R, Meyer T *et al*: Point mutations of the p150 subunit of dynactin (DCTN1) gene in ALS. *Neurology* 2004; **63:** 724-726.

[9] OMIM: Online Mendelian Inheritance in Man, OMIM (TM): Available at. http://www.ncbi.nlm.nih.gov/omim/. Accessed February 2014.

[10] Capell BC, Collins FS: Human laminopathies: nuclei gone genetically awry. *Nat Rev Genet* 2006; **7:** 940-952.

[11] Rankin J, Auer-Grumbach M, Bagg W *et al*: Extreme phenotypic diversity and nonpenetrance in families with the LMNA gene mutation R644C. *Am J Med Genet A* 2008; **146A:** 1530-1542.

[12] Mercuri E, Brown SC, Nihoyannopoulos P *et al*: Extreme variability of skeletal and cardiac muscle involvement in patients with mutations in exon 11 of the lamin A/C gene. *Muscle & nerve* 2005; **31:** 602-609.

[13] Muntoni F, Bonne G, Goldfarb LG *et al*: Disease severity in dominant Emery Dreifuss is increased by mutations in both emerin and desmin proteins. *Brain* 2006; **129:** 1260-1268.

[14] Hayashi A, Kasahara T, Iwamoto K *et al*: The role of brain-derived neurotrophic factor (BDNF)-induced XBP1 splicing during brain development. *J Biol Chem* 2007; **282:** 34525-34534.

[15] Verhoeven K, De Jonghe P, Van de Putte T *et al*: Slowed conduction and thin myelination of peripheral nerves associated with mutant rho Guanine-nucleotide exchange factor 10. *Am J Hum Genet* 2003; **73:** 926-932.

[16] De Sandre-Giovannoli A, Chaouch M, Kozlov S *et al*: Homozygous defects in LMNA, encoding lamin A/C nuclear-envelope proteins, cause autosomal recessive axonal neuropathy in human (Charcot-Marie-Tooth disorder type 2) and mouse. *Am J Hum Genet* 2002; **70:** 726-736.

[17] Winterthun S, Ferrari G, He L *et al*: Autosomal recessive mitochondrial ataxic syndrome due to mitochondrial polymerase gamma mutations. *Neurology* 2005; **64:** 1204-1208.

[18] Lax NZ, Whittaker RG, Hepplewhite PD *et al*: Sensory neuronopathy in patients harbouring recessive polymerase gamma mutations. *Brain* 2012; **135:** 62-71.

[19] Van Goethem G, Luoma P, Rantamaki M *et al*: POLG mutations in neurodegenerative disorders with ataxia but no muscle involvement. *Neurology* 2004; **63:** 1251-1257.

[20] Harrower T, Stewart JD, Hudson G *et al*: POLG1 mutations manifesting as autosomal recessive axonal Charcot-Marie-Tooth disease. *Arch Neurol* 2008; **65:** 133-136.

[21] Zemmouri R, Azzedine H, Assami S *et al*: Charcot-Marie-Tooth 2-like presentation of an Algerian family with giant axonal neuropathy. *Neuromuscul Disord* 2000; **10:** 592-598.

[22] Xu M, Da YW, Liu L, Wang F, Jia JP: Giant axonal neuropathy caused by a novel compound heterozygous mutation in the gigaxonin gene. *J Child Neurol* 2013; **28:** 1316-1319.

[23] Houlden H, Groves M, Miedzybrodzka Z *et al*: New mutations, genotype phenotype studies and manifesting carriers in giant axonal neuropathy. *J Neurol Neurosurg Psychiatry* 2007; **78:** 1267-1270.

[24] Bomont P, Cavalier L, Blondeau F *et al*: The gene encoding gigaxonin, a new member of the cytoskeletal BTB/kelch repeat family, is mutated in giant axonal neuropathy. *Nat Genet* 2000; **26:** 370-374.

[25] Kuhlenbaumer G, Young P, Oberwittler C *et al*: Giant axonal neuropathy (GAN): case report and two novel mutations in the gigaxonin gene. *Neurology* 2002; **58:** 1273-1276.

[26] Demir E, Bomont P, Erdem S *et al*: Giant axonal neuropathy: clinical and genetic study in six cases. *J Neurol Neurosurg Psychiatry* 2005; **76:** 825-832.

[27] Bomont P, Ioos C, Yalcinkaya C *et al*: Identification of seven novel mutations in the GAN gene. *Hum Mutat* 2003; **21:** 446.

[28] Baets J, Deconinck T, De Vriendt E *et al*: Genetic spectrum of hereditary neuropathies with onset in the first year of life. *Brain* 2011; **134:** 2664-2676.

[29] Brockmann K, Dreha-Kulaczewski S, Dechent P *et al*: Cerebral involvement in axonal Charcot-Marie-Tooth neuropathy caused by mitofusin2 mutations. *J Neurol* 2008; **255:** 1049-1058.

[30] Genari AB, Borghetti VH, Gouvea SP *et al*: Characterizing the phenotypic manifestations of MFN2 R104W mutation in Charcot-Marie-Tooth type 2. *Neuromuscul Disord* 2011; **21:** 428-432.

[31] Almeida-Souza L, Goethals S, de Winter V *et al*: Increased monomerization of mutant HSPB1 leads to protein hyperactivity in Charcot-Marie-Tooth neuropathy. *J Biol Chem* 2010; **285:** 12778-12786.

[32] Almeida-Souza L, Asselbergh B, d'Ydewalle C *et al*: Small heat-shock protein HSPB1 mutants stabilize microtubules in Charcot-Marie-Tooth neuropathy. *J Neurosci* 2011; **31:** 15320-15328.

[33] Evgrafov OV, Mersiyanova I, Irobi J *et al*: Mutant small heat-shock protein 27 causes axonal Charcot-Marie-Tooth disease and distal hereditary motor neuropathy. *Nat Genet* 2004; **36:** 602-606.

[34] Solla P, Vannelli A, Bolino A *et al*: Heat shock protein 27 R127W mutation: evidence of a continuum between axonal Charcot-Marie-Tooth and distal hereditary motor neuropathy. *J Neurol Neurosurg Psychiatry* 2010; **81:** 958-962.

[35] Bruno C, Bertini E, Federico A *et al*: Clinical and molecular findings in patients with giant axonal neuropathy (GAN). *Neurology* 2004; **62:** 13-16.

[36] Zhao C, Takita J, Tanaka Y *et al*: Charcot-Marie-Tooth disease type 2A caused by mutation in a microtubule motor KIF1Bbeta. *Cell* 2001; **105:** 587-597.

[37] Horvath R, Hudson G, Ferrari G *et al*: Phenotypic spectrum associated with mutations of the mitochondrial polymerase gamma gene. *Brain* 2006; **129:** 1674-1684.

[38] Hinnell C, Haider S, Delamont S, Clough C, Hadzic N, Samuel M: Dystonia in mitochondrial spinocerebellar ataxia and epilepsy syndrome associated with novel recessive POLG mutations. *Mov Disord* 2012; **27:** 162-163.

[39] Senderek J, Bergmann C, Stendel C *et al*: Mutations in a gene encoding a novel SH3/TPR domain protein cause autosomal recessive Charcot-Marie-Tooth type 4C neuropathy. *Am J Hum Genet* 2003; **73:** 1106-1119.

[40] Azzedine H, Ravise N, Verny C *et al*: Spine deformities in Charcot-Marie-Tooth 4C caused by SH3TC2 gene mutations. *Neurology* 2006; **67:** 602-606.

[41] Gosselin I, Thiffault I, Tetreault M *et al*: Founder SH3TC2 mutations are responsible for a CMT4C French-Canadians cluster. *Neuromuscul Disord* 2008; **18:** 483-492.

[42] Houlden H, Laura M, Ginsberg L *et al*: The phenotype of Charcot-Marie-Tooth disease type 4C due to SH3TC2 mutations and possible predisposition to an inflammatory neuropathy. *Neuromuscul Disord* 2009; **19:** 264-269.

[43] Lupski JR, Reid JG, Gonzaga-Jauregui C *et al*: Whole-genome sequencing in a patient with Charcot-Marie-Tooth neuropathy. *N Engl J Med* 2010; **362:** 1181-1191.

[44] Weedon MN, Hastings R, Caswell R *et al*: Exome sequencing identifies a DYNC1H1 mutation in a large pedigree with dominant axonal Charcot-Marie-Tooth disease. *Am J Hum Genet* 2011; **89:** 308-312.

[45] Bolino A, Lonie LJ, Zimmer M *et al*: Denaturing high-performance liquid chromatography of the myotubularin-related 2 gene (MTMR2) in unrelated patients with Charcot-Marie-Tooth disease suggests a low frequency of mutation in inherited neuropathy. *Neurogenetics* 2001; **3:** 107-109.

[46] Parman Y, Battaloglu E, Baris I *et al*: Clinicopathological and genetic study of early-onset demyelinating neuropathy. *Brain* 2004; **127:** 2540-2550.

[47] Nelis E, Erdem S, Tan E *et al*: A novel homozygous missense mutation in the myotubularin-related protein 2 gene associated with recessive Charcot-Marie-Tooth disease with irregularly folded myelin sheaths. *Neuromuscul Disord* 2002; **12:** 869-873.

[48] Chaya T, Shibata S, Tokuhara Y *et al*: Identification of a negative regulatory region for the exchange activity and characterization of T332I mutant of Rho guanine nucleotide exchange factor 10 (ARHGEF10). *J Biol Chem* 2011; **286:** 29511-29520.

[49] Ng PC, Henikoff S: Predicting deleterious amino acid substitutions. *Genome Res* 2001; **11:** 863-874.

[50] Adzhubei IA, Schmidt S, Peshkin L *et al*: A method and server for predicting damaging missense mutations. *Nature methods* 2010; **7:** 248-249.

[51] Hicks S, Wheeler DA, Plon SE, Kimmel M: Prediction of missense mutation functionality depends on both the algorithm and sequence alignment employed. *Hum Mutat* 2011; **32:** 661-668.

[52] Schwarz JM, Rodelsperger C, Schuelke M, Seelow D: MutationTaster evaluates disease-causing potential of sequence alterations. *Nature methods* 2010; **7:** 575-576.

[53] Li AD: SpliceSiteFinder: Available at. http://www.genet.sickkids.on.ca/~ali/splicesitefinder.html.

[54] Yeo G, Burge CB: Maximum entropy modeling of short sequence motifs with applications to RNA splicing signals. *J comput biol* 2004; **11:** 377-394.

[55] Reese MG, Eeckman FH, Kulp D, Haussler D: Improved splice site detection in Genie. *J comput biol* 1997; **4:** 311-323.

[56] Pertea M, Lin X, Salzberg SL: GeneSplicer: a new computational method for splice site prediction. *Nucleic Acids Res* 2001; **29:** 1185-1190.

[57] Desmet FO, Hamroun D, Lalande M, Collod-Beroud G, Claustres M, Beroud C: Human Splicing Finder: an online bioinformatics tool to predict splicing signals. *Nucleic Acids Res* 2009; **37:** e67.

[58] Franceschini A, Szklarczyk D, Frankild S *et al*: STRING v9.1: protein-protein interaction networks, with increased coverage and integration. *Nucleic Acids Res* 2013; **41:** D808-815.

[59] Ingenuity®Systems: Ingenuity Pathway Analysis.

[60] Matthews L, Gopinath G, Gillespie M *et al*: Reactome knowledgebase of human biological pathways and processes. *Nucleic Acids Res* 2009; **37:** D619-622.
